# Genome-Wide Identification of the *GbUBC* Gene Family in Sea-Island Cotton (*Gossypium barbadense*) and the Active Regulation of Drought Resistance in Cotton by *GbUBC23*

**DOI:** 10.3390/ijms252312948

**Published:** 2024-12-02

**Authors:** Yi Wang, Zheng Zong, Junchen Chen, Xue Sun, Jiahui Wang, Yuehua Yu, Zhiyong Ni

**Affiliations:** 1 Key Laboratory of Ecological Adaptation and Evolution of Extreme Environment in Xinjiang, College of Life Sciences, Xinjiang Agricultural University, Urumqi 830052, China; wangyi604987664@126.com (Y.W.); z19112919547@163.com (Z.Z.); chenjunchen9248@163.com (J.C.); 18793520236@163.com (X.S.); wangjh22200@163.com (J.W.); 2 College of Agronomy, Xinjiang Agricultural University, Urumqi 830052, China

**Keywords:** ubiquitination, *GbUBC* gene family, *GbUBC23*, drought

## Abstract

Cotton is an economically critical crop worldwide, and drought stress strongly affects its growth and development. Ubiquitination modifies protein activity and is crucial in numerous biological processes. Ubiquitin-conjugating enzymes serve as intermediaries in the protein ubiquitination process and play important roles in plant responses to abiotic stress. However, the impact of ubiquitination on the response of cotton to abiotic stress is not fully understood. Bioinformatic methods were employed in this study to analyze the physiochemical characteristics, gene structure, collinearity, expression patterns, and evolutionary relationships of GbUBC gene family members in sea-island cotton. In sea-island cotton, a minimum of 125 GbUBC genes are irregularly distributed across the 26 chromosomes, with multiple instances of gene duplication observed among the members. Phylogenetic analysis categorized the GbUBC gene family into 15 ubiquitin-conjugating enzyme (E2) subgroups, one ubiquitin E2 enzyme variant (UEV) subgroup, and one COP10 subgroup. GbUBC gene expression pattern analyses revealed that most GbUBC genes responded differently to cold, heat, NaCl, and polyethylene glycol (PEG) treatments, with certain GbUBC genes exhibiting high expression levels in specific fiber development period and organs. Furthermore, molecular biology methods were employed to elucidate the biological functions of GbUBC23. The GbUBC23 gene was highly expressed in the cotyledons of sea-island cotton and was activated by PEG treatment. GbUBC23 is localized to the nucleus and cytomembrane. The silencing of the GbUBC23 gene under drought conditions led to decreased drought tolerance and survival rates in sea-island cotton. Compared with those in the control plants, the activity of proline and superoxide dismutase and the expression levels of the drought-induced genes GbNCED3, GbRD22, GbRD26 were significantly lower, but the levels of malondialdehyde and hydrogen peroxide were significantly higher. Our findings revealed 125 members of the GbUBC gene family in sea-island cotton, with the GbUBC23 gene critically contributing to the abiotic stress response. These findings indicate that the GbUBC gene family may play a crucial role in the drought stress response in sea-island cotton.

## 1. Introduction

One of the most common posttranslational modifications to proteins, ubiquitination, is essential for selective protein breakdown in plants [1,2,3]. The ubiquitination process is coordinated by three key enzymes: ubiquitin-activating enzymes (E1s), ubiquitin-conjugating enzymes (E2s), and ubiquitin-ligating enzymes (E3s) [4]. This system labels the target protein with a degradation signal through the ubiquitination process and then degrades this protein in the 26S proteasome. E2s are crucial enzymes in this process, serving as intermediaries for delivering active ubiquitin to E3s or directly to the target protein [5]. The functions of E2s in plants concerning the regulation of responses to biotic and abiotic stressors, hormone signaling, and growth and development have been progressively elucidated [6]. Therefore, an in-depth study of the function and mechanism of E2s is highly important for understanding cell biology and plant physiology.

Members of the E2 family are classified on the basis of their conserved domains and functional properties. E2s typically contain a highly conserved ubiquitin-conjugating (UBC) domain of approximately 150 amino acids containing a catalytic cysteine residue (Cys) that serves asthe active site in ubiquitin transfer. In contrast, ubiquitin E2 enzyme variants (UEVs) lack this Cys and usually act as cofactors of active E2s [1,7,8]. The *GbUBC* gene family can be classified into UBC and UEV groups on the basis of variations in amino acid sequence and domain structure.

To date, multiple UBC family genes have been identified in *Arabidopsis* [5], upland cotton (*Gossypium hirsutum*) [9], wheat (*Triticum aestivum*) [10], papaya (*Carica papaya*) [11], longan (*Dimocarpus longan*) [12], grape (*Vitis vinifera*) [13], rice (*Oryza sativa*) [14], sorghum (*Sorghum bicolor*) [15], potato (*Solanum tuberosum*) [16], tomato (*Solanum lycopersicum*) [17], bananas (*Musa acuminata*) [18], soybean (*Glycine max*) [19], maize (*Zea mays*) [20], and *Brassica napus* [21]. In addition, some *UBC* genes have been functionally validated in plants. The *UBC* genes play crucial roles in *Arabidopsis* flowering [22], seedling photomorphogenesis [23], root hair development [24,25], and ovule growth [26]. *SlUBC32* is a regulatory factor for fruit ripening in tomatoes [27]. *GhUBC2L* regulates the organ size of upland cotton through histone monoubiquitination [9]. Furthermore, *UBC* genes are involved in plant responses to various environmental stressors, such as drought, salt, high temperature, and low temperature. *UBC13* participates in the response of *Arabidopsis* to low-temperature stress [28]. UBC15 and its complexes mediate the degradation of the Short Vegetative Phase (SVP) at high temperatures, accelerating *Arabidopsis* flowering [29]. The mutation of *AtUBC32* can increase salt tolerance in *Arabidopsis* [30]. The silencing of the *AtUBC32*, *AtUBC33*, and *AtUBC34* genes in *Arabidopsis* resulted in stomatal closure and increased drought stress tolerance [31]. *GmUBC9* overexpression increased drought tolerance in both soybean and *Arabidopsis* [19]. However, in cotton, there is only one example of the involvement of the *UBC* gene in abiotic stress. In upland cotton, GhUBC10-2 facilitates the degradation of GhGSTU17 to modulate salt tolerance [32]. Consequently, *UBC* genes are necessary for the growth and development of plants, as well as their reactions to abiotic stress from the environment.

Cotton is a critical source of fiber for the textile sector. Nevertheless, research on UBC family members and abiotic resistance-related genes in sea-island cotton has been limited. To gain a better understanding of the role that *GbUBC* genes play in the growth and development of sea-island cotton as well as their reactions to abiotic stress, biological information techniques were employed to identify the *GbUBC* genes, and the structure and function, physical and chemical properties, chromosome localization, subcellular localization, stress heatmaps, different stages of fiber development and organ heatmaps, and phylogenetic relationships among the family members were systematically analyzed. The function of the ubiquitin-conjugating enzyme GbUBC23 was determined. Silencing the *GbUBC23* gene in sea-island cotton significantly decreased the drought tolerance phenotype, the survival rate, and the water-loss rate, reduced proline levels and superoxide dismutase (SOD) activity, and elevated malondialdehyde (MDA) and hydrogen peroxide (H_2_O_2_) levels. This study provides a theoretical framework for examining the molecular processes via which *GbUBC* genes respond to drought stress in sea-island cotton, identifying a new candidate gene for the development of drought-resistant cultivars of this crop.

## 2. Results

### 2.1. Identification of the UBC Gene Family in Sea-Island Cotton

A total of 303 GbUBC protein sequences were examined in this investigation. After manually removing proteins without the UBC domain, deleting short non-full-length fragments, and removing superfluous sequences of identical genes, we obtained 125 matching gene sequences, and these 125 genes were considered the *GbUBC* genes of sea-island cotton (Table S1). Furthermore, we named the 125 sea-island cotton *UBC* genes *GbUBC1*–*GbUBC125* according to their location in the genome.

The gene name, gene locus, amino acid size, molecular weight (MW), theoretical isoelectric point (PI), aliphatic index, grand average of hydropathicity, and subcellular localization prediction information for all *UBC* gene families of sea-island cotton are provided in Table S2. The coding region lengths of each of the genes were between 339 bp (*GbUBC38*) and 3345 bp (*GbUBC84*), and the MWs of the coding proteins varied from 12.54 kDa (GbUBC38) to 123.4 kDa (GbUBC84). The pIs ranged from 4.21 (GbUBC97) to 9.49 (GbUBC110), and the aliphatic indices ranged from 65.65 (GbUBC71) to 95.1 (GbUBC74). Subcellular localization predictions revealed that 104 GbUBC proteins are located to the nucleus, 8 to the cytoplasm, 1 to the endoplasmic reticulum, 10 to the nucleus and cytoplasm, and 2 to the chloroplasts and nucleus.

### 2.2. Chromosome Localization and Phylogenetic Analysis of the GbUBC Genes

**Figure 1:**
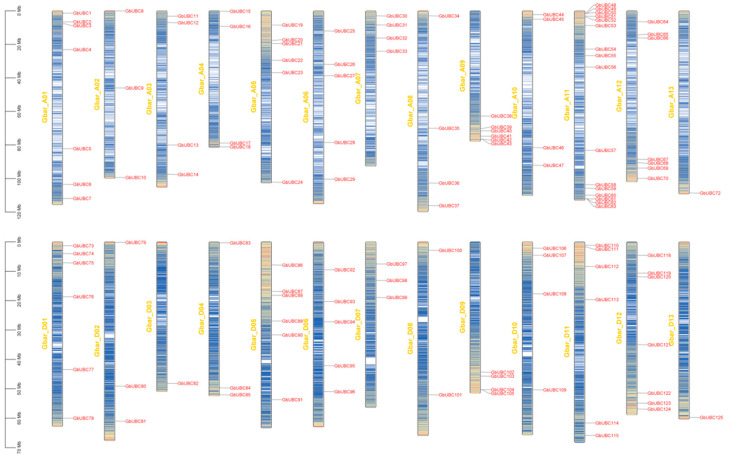
Collinearity and chromosome localization analysis of the *GbUBCs*. The heatmap represents chromosome density. The color scale indicates low density (blue) and high density (red).

Sea-island cotton has 125 *GbUBC* genes dispersed unevenly over the 26 chromosomes (Figure 1), with 72 and 53 genes in the chromosomal A and D groups, respectively. Chromosome A11 has the most UBC genes, with 16; chromosomes A13, D03, and D13 each contain one UBC gene; two UBC genes are located on chromosome D08; chromosomes A02, D02, D04, and D07 each contain three UBC genes; chromosomes A03, A04, A07, A08, A10, D09, and D10 all contain four UBC genes; chromosomes A06 and D06 both possess five UBC genes; chromosomes A05, A09, D01, and D05 all contain six UBC genes; chromosomes A01 and D12 both contain seven UBC genes; and chromosomes A12 and D11 each contain eight UBC genes.

**Figure 2:**
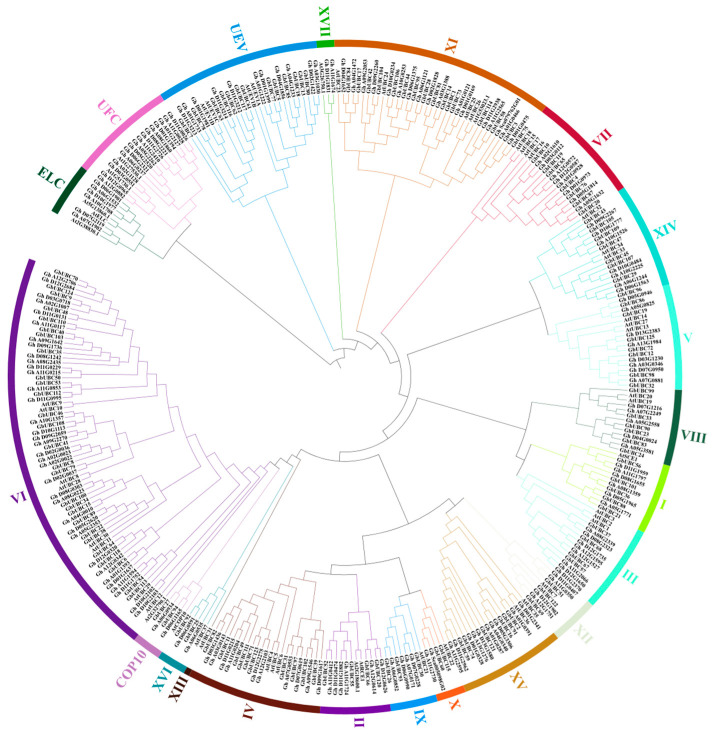
Phylogenetic relationships among the UBC proteins. Phylogenetic analysis was performed on *Arabidopsis* (53), *G. hirsutum* (169), and *Gossypium barbadense* (125) via MEGA6.06 software. Different colors represent various subfamilies of the UBC gene family. The UBC gene family comprises 21 subgroups, each designated by its respective name.

We developed a phylogenetic tree to examine the evolutionary relationships of the *GbUBC* genes via UBC sequences of proteins from sea-island cotton, upland cotton, and *Arabidopsis*. The phylogenetic tree (Figure 2) divided the *GbUBC* gene family into 21 different groups, including typical UBC groups I-XVI and atypical UBC groups such as UEV, UFC, ELC, COP10, and XVII. Among them, the VI group was the largest branch among all groups, with a total of 76 members. In addition, we identified 29 *GbUBC* gene family members in group VI, accounting for 38.2% of the group’s members. In the entire evolutionary tree, group VI also has the most *GbUBC* gene members; no *GbUBC* gene members are present in subfamilies XIII, XVII, UFC, and ELC. Additionally, we found that the UBC genes of three species are present in almost every branch. These findings indicate that the evolutionary history of the *GbUBC* gene members aligns with those of *Arabidopsis* and upland cotton. These findings suggest that the UBC genes may have rapidly multiplied before sea-island cotton diverged from upland cotton and *Arabidopsis*.

### 2.3. Structural Analysis of the GbUBC Genes

**Figure 3:**
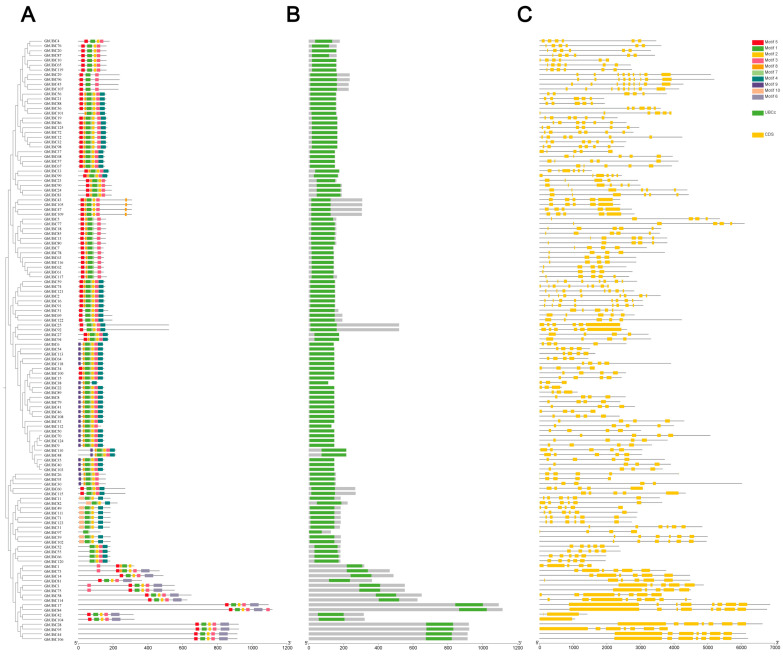
Structural distribution patterns of the 125 *GbUBC* genes. (**A**) MEME conserved motif distribution. The colored boxes denote various conserved motifs. (**B**) Distribution of the conserved domain UBCc. The green square represents the conserved domain of UBCc. (**C**) *GbUBC* gene structure, with exons depicted as yellow boxes.

An analysis of conserved motifs was performed on 125 GbUBC protein sequences in sea-island cotton via the MEME online program, resulting in the prediction of 10 motifs (Figure 3A). The distribution and quantity of motifs in the GbUBC proteins indicate that each protein contains between two and seven motifs. All 125 proteins share the common motif 1, and most sequences also contain motifs 2 and 3 (Figure 3A). These findings suggest that these motifs are important for the function and conserved position of the UBC proteins. Moreover, we found that within each group, the majority of GbUBC members had similar motif compositions, while there were significant differences in motif compositions between different groups (Figure 3A). The UBC domain corresponding to the motif is also shown in Figure 3B. The results show that the UBC gene family proteins are categorized into distinct subgroups on the basis of different UBC domains, each serving different functions.

During the evolution of gene families, the introns and exons served distinct functions in gene expression and regulation. The quantity and distribution of introns and exons in the *GbUBC* gene family in sea-island cotton demonstrate its evolutionary relationship with the genome. A significant correlation was observed among the structures of gene family members throughout the evolutionary process, with clustered members exhibiting similar structural characteristics. The number of exons in the *GbUBC* gene family in sea-island cotton ranged from 1 to 9, whereas the number of introns ranged from 0 to 10 (Figure 3C). Among them, *GbUBC103* does not contain any introns, whereas *GbUBC28* and *GbUBC105* contain 10 introns; *GbUBC29*, *GbUBC45*, *GbUBC95*, and *GbUBC106* contain nine exons, which is the greatest number of exons; and *GbUBC42* and *GbUBC104* contain only one exon, which is the smallest number (Figure 3C). Most members contained five (26.40%) or six (29.60%) exons (Figure 3C). This finding indicates their conservation during evolution and suggests that these genes may have similar functions.

### 2.4. Analysis of the Cis-Acting Elements of the GbUBC Genes

**Figure 4:**
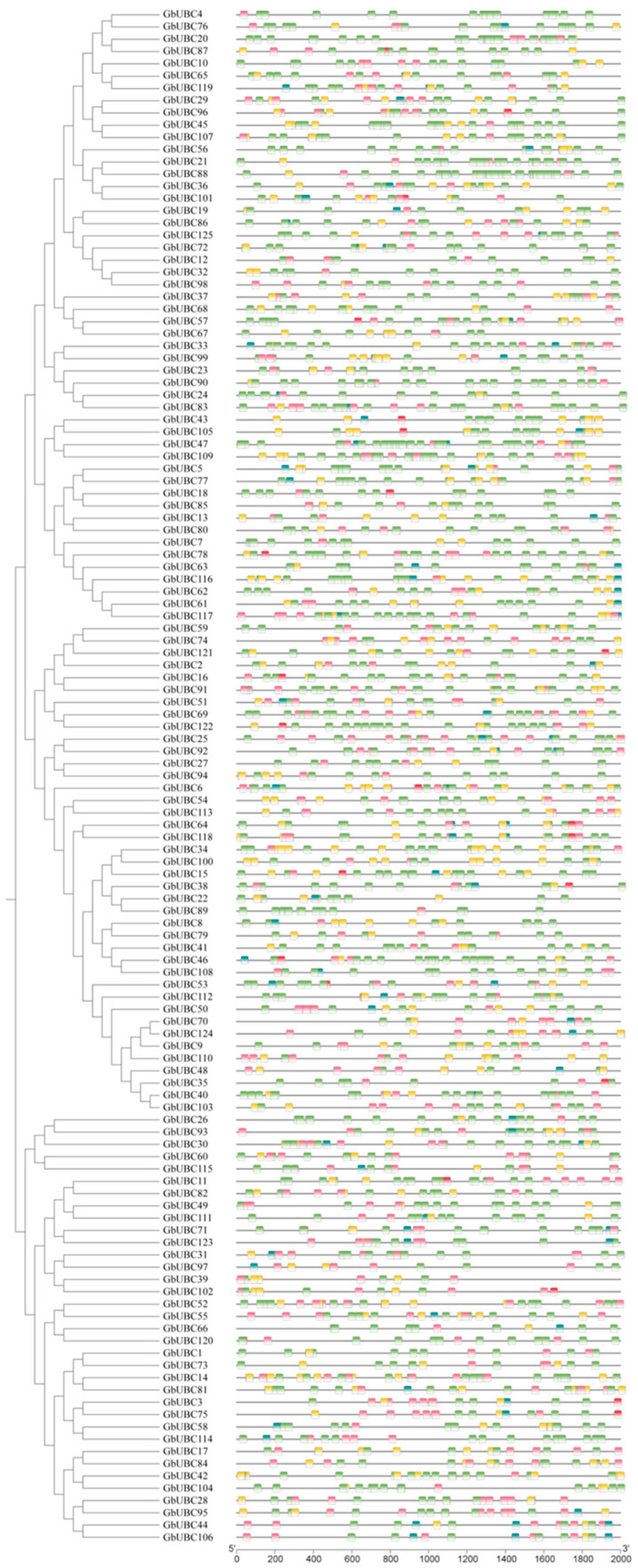
*Cis*-acting elements of the *GbUBC* genes in sea-island cotton. Five groups of *cis*-acting elements were identified in the promoter regions of the *GbUBC* genes, each represented by distinct colored boxes. Green boxes denote *cis*-acting elements associated with the light response, yellow boxes indicate *cis*-acting elements linked to hormones and chemical reactions, pink boxes signify *cis*-acting elements related to the abiotic stress response, blue-green boxes represent *cis*-acting elements related to growth, and red boxes represent *cis*-acting elements connected to diurnal cycles.

*Cis*-acting elements in the promoter region regulate gene transcription and other biological processes. The 2 kb area upstream of the transcription start site of the *GbUBC* genes included 41 unique *cis*-acting elements. The PlantCARE functional annotation categorizes the results into five groups: *cis*-acting elements linked to the abiotic stress response (ARE, MBS, LTR, etc.), *cis*-acting elements related to hormonal and chemical responses (ABRE, P-box, CGTCA motif, etc.), *cis*-acting elements pertaining to circadian rhythms (circular), *cis*-acting elements associated with the light response (Box4, GT1 motif, G-box, etc.), and *cis*-acting elements connected to growth (CAT box, GCN4 motif, etc.). The most common *cis*-acting element (1622) is associated with the light response. Second, 575 *cis*-acting elements are associated with hormones and chemical reactions, 485 are associated with abiotic stress, 94 are associated with growth, and the fewest (27) are associated with the circadian rhythm. Additionally, various *cis*-acting elements related to the drought stress response were identified, including MBS (drought induction capability), ABRE (*cis*-active element associated with the abscisic acid response), TCA (*cis*-active element linked to the salicylic acid response), TGACG (*cis*-active regulatory element related to MeJA responsiveness), and TGA (auxin-responsive element) (Figure 4).

### 2.5. Gene Duplication and Collinearity Analysis of the GbUBC Gene Family

**Figure 5:**
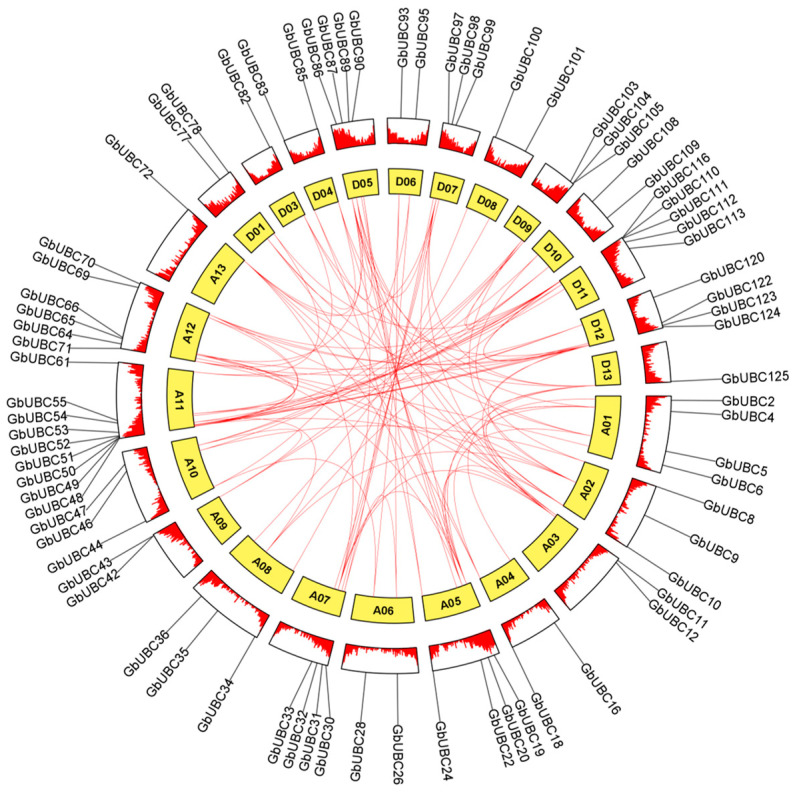
Distribution of chromosomal *GbUBC* segment duplication gene pairs in sea-island cotton. Duplicated gene pairs are marked by red lines. The yellow blocks signify chromosomes. The chromosome number is indicated on the inner surface of each chromosome.

We analyzed tandem and large-scale segmental duplication events in the *GbUBC* gene family during evolution. The results revealed that 79.2% (99/125) of the *GbUBC* genes were the result of duplication events, including three pairs of tandemly duplicated genes (*GbUBC61*/*GbUBC62*, *GbUBC62*/*GbUBC63*, and *GbUBC116*/*GbUBC117*). These genes are located on chromosomes A11 and D11, whereas the remaining genes are segmental duplicates (Figure 5). These findings suggest that segmental duplication events may have primarily driven the formation and expansion of the *GbUBC* gene family. To increase our comprehension of the evolutionary selection pressures on *GbUBC* genes, we computed the Ka/Ks ratios of the duplicated genes. The findings indicated that among 101 duplicated gene pairs, two pairs (*GbUBC19*/*GbUBC86* and *GbUBC42*/*GbUBC104*) presented Ka/Ks ratios exceeding 1, indicating positive selection and that evolution had altered the proteins (Table S3). Two pairs of *GbUBC* genes with Ka/Ks values of NaN were omitted from the analysis. The fact that the Ka/Ks values for the remaining 97 gene pairs were all lower than one (Table S3) indicates that these genes have been subjected to purifying selection. The genes in the *GbUBC* gene family in sea-island cotton underwent relatively rapid evolution after chromosome segmental duplication; thus, the *GbUBC* genes are functionally separated and participate in various physiological activities.

### 2.6. Expression Analysis of the GbUBC Genes

**Figure 6:**
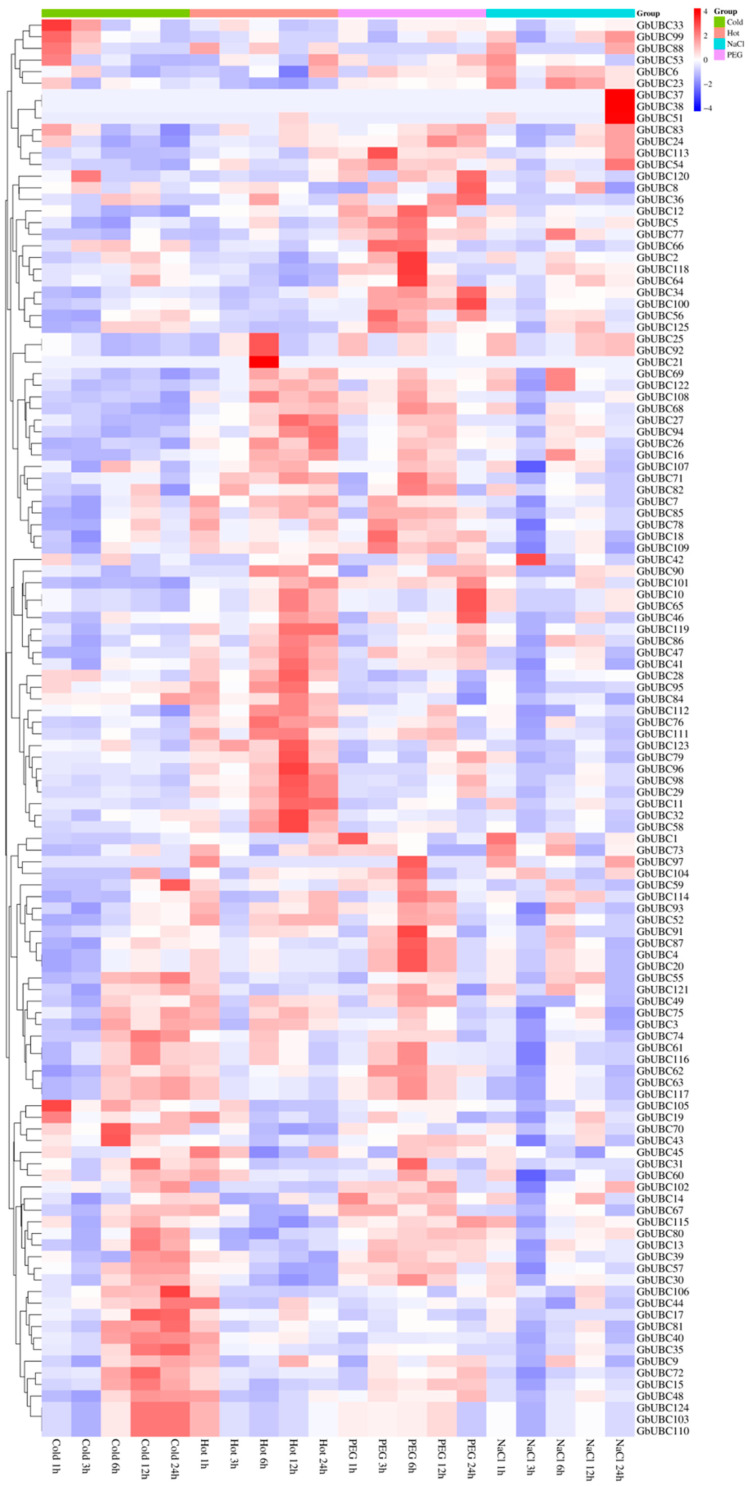
Expression analysis of the *GbUBC* genes under abiotic stress conditions. The heatmap presents an illustration of the evolutionary grouping of the 125 genes belonging to the *GbUBC* gene family. Blue represents low expression, whereas red indicates high expression.

RNA sequencing data from the cotton expression database were used to examine *GbUBC* gene expression in sea-island cotton. The analysis included different abiotic stresses (cold treatment, heat treatment, drought stress, and salt stress) and various developmental stages. The results indicated that almost all the *GbUBC* genes were expressed under different abiotic stresses, except for three genes (*GbUBC22*, *GbUBC50*, and *GbUBC89*), whose expression was not affected by abiotic stress (Figure 6). Members of the same group presented similar expression patterns, such as *GbUBC9*/*GbUBC15*/*GbUBC35*/*GbUBC40*/*GbUBC48*/*GbUBC70*/*GbUBC103*/*GbUBC110*/*GbUBC124* in subgroup VI and *GbUBC14*/*GbUBC17*/*GbUBC44*/*GbUBC81*/*GbUBC106* in subgroup XI, which presented relatively high expression levels during cold treatment (Figure 6). Similarly, the expression levels of *GbUBC4*/*GbUBC10*/*GbUBC20*/*GbUBC65*/*GbUBC87* in group VII and *GbUBC61*/*GbUBC62*/*GbUBC63*/*GbUBC77*/*GbUBC116*/*GbUBC117* in the UEV group were relatively high under PEG stress (Figure 6).

**Figure 7:**
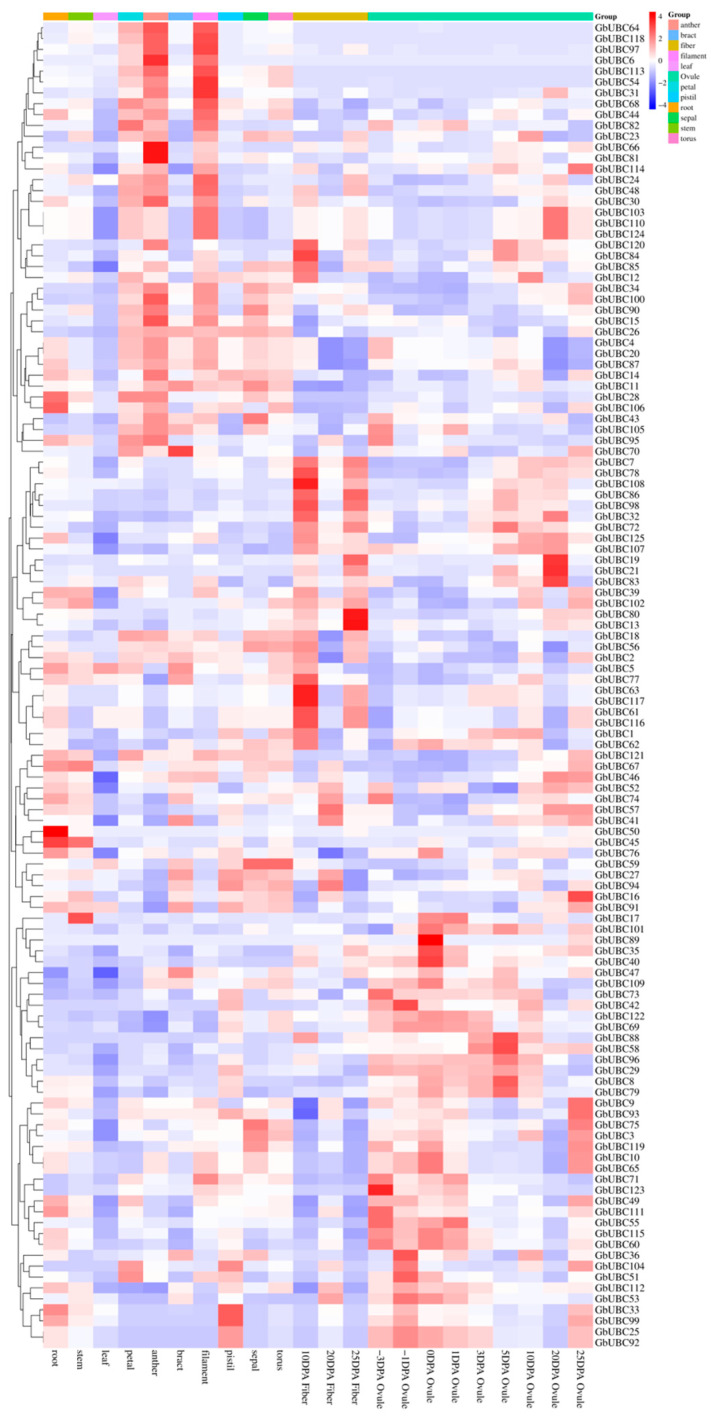
Expression analysis of the *GbUBC* genes in different organs and at different stages of fiber development. The heatmap illustrates the phylogenetic clustering of the 125 *GbUBC* genes. Blue signifies low expression, whereas red denotes high expression.

The expression levels of the *GbUBC* gene family were investigated in various organs and at various stages of fiber development. The findings revealed that most *GbUBC* genes were expressed across different organs, whereas only three genes (*GbUBC22*, *GbUBC37*, and *GbUBC38*) presented no expression in any organ (Figure 7). Furthermore, within each organ, the expression levels of genes belonging to the same groups were found to be similar. For example, genes such as *GbUBC6*/*GbUBC54*/*GbUBC64*/*GbUBC113*/*GbUBC118* in group VI presented high expression levels in the petals, filaments, and anthers (Figure 7). Similarly, during the same period of fiber development, the expression levels of genes belonging to the same group were also similar. For example, the expression levels of genes such as GbUBC19/GbUBC32/GbUBC72/GbUBC86/GbUBC98/GbUBC125 in group V and GbUBC7/GbUBC78/GbUBC80/GbUBC13/GbUBC117/GbUBC61/GbUBC116/GbUBC62 in the UEV group were relatively high in cotton fibers at 10 and 25 days post-anthesis (DPA). The expression levels of the GbUBC71/GbUBC123/GbUBC49/GbUBC111 genes in group IV were greater at −3 DPA in the ovule.

The expression analysis revealed that the *GbUBC* genes in sea-island cotton are highly expressed under various abiotic stresses. Some genes are strongly expressed under numerous abiotic challenges, suggesting that the *GbUBC* genes of sea-island cotton may respond to various abiotic stressors. The *GbUBC* genes in several organs of sea-island cotton do not demonstrate notable expression selectivity. Aside from a limited number of genes that exhibit strong expression in certain organs, the majority of genes are expressed throughout several organs. These results suggest that the *GbUBC* genes in sea-island cotton may be involved in regulating several phases of cotton growth and development, as well as in reactions to diverse abiotic stimuli.

### 2.7. GbUBC23 Is Involved in Drought Stress and Is Localized to the Nucleus and Cell Membrane

**Figure 8:**
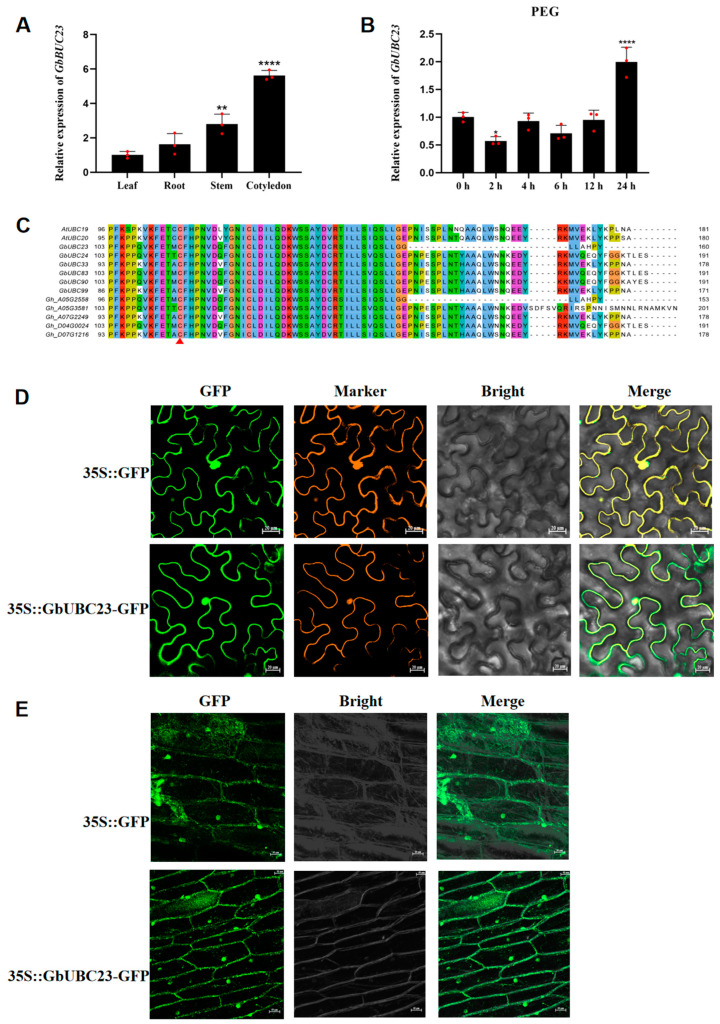
*GbUBC23* is involved in drought stress and is localized to the nucleus and cell membrane. (**A**) Expression profile of *GbUBC23* in different cotton organs; (**B**) expression profile of *GbUBC23* 24 h after PEG stress; (**C**) sequence alignment of the GbUBC23 protein and its homologs in sea-island cotton, upland cotton, and *Arabidopsis*. The red triangle shows the conserved catalytic cysteine residue; (**D**) subcellular GbUBC23 localization in *N. benthamiana*. Marker: red fluorescent protein and calcineurin B-like protein, nuclear and membrane marker; GFP: green fluorescence protein; Bright: visible light; Merge: merged bright with Marker and GFP; (**E**) subcellular GbUBC23 localization in onion cells. GFP: green fluorescence protein; Bright: visible light; Merge: merged bright with Marker and GFP. The vertical bars denote the means ± standard deviations, with significant differences relative to leaves and 0 h indicated by ** *p* < 0.001 and **** *p* < 0.0001.

We studied *GbUBC23* in sea-island cotton to further elucidate the possible roles of *GbUBC* gene family members. *GbUBC23* was substantially expressed in sea-island cotton cotyledons (Figure 8A) and was also highly expressed 24 h after PEG stress (Figure 8B). The *GbUBC23* gene encodes a 161-amino acid UBC protein, and the members of subgroup VIII in *Arabidopsis*, sea-island cotton, and upland cotton all have high identity and all contain conserved cysteine residues, which are typical of UBC proteins (Figure 8C). These findings suggest that the sea-island cotton *GbUBC23* gene arose from the same ancestry of genes.

We utilized Plant-mPLoc (http://www.csbio.sjtu.edu.cn/bioinf/plant-multi/) (accessed on 26 November 2024) to predict the subcellular localization of GbUBC23, aiming to increase our understanding of its molecular mechanism. The Plant-mPLoc results revealed that GbUBC23 was localized to the nucleus. To further confirm these findings, we injected 35S::GbUBC23-GFP into *N. benthamiana* to evaluate the subcellular localization of GbUBC23, with 35S::GFP used as a control. Two types of markers were utilized: membrane localization signal-tagged and nuclear localization signal-tagged. GFP was uniformly distributed in *N. benthamiana* leaf mesophyll cells, whereas 35S::GbUBC23-GFP was confined to the nucleus and cell membrane (Figure 8D). To validate the subcellular localization results in *N. benthamiana* cells, we employed GFP-tagged protein expression vectors to transform onion epidermal cells. The subcellular localization analysis in onion cells revealed that both GFP and GbUBC23-GFP were localized to the nucleus and cell membrane (Figure 8E), providing further support for the subcellular localization results in *N. benthamiana*.

### 2.8. GbUBC23 Gene Silencing Decreases the Tolerance of Sea-Island Cotton to Drought

**Figure 9:**
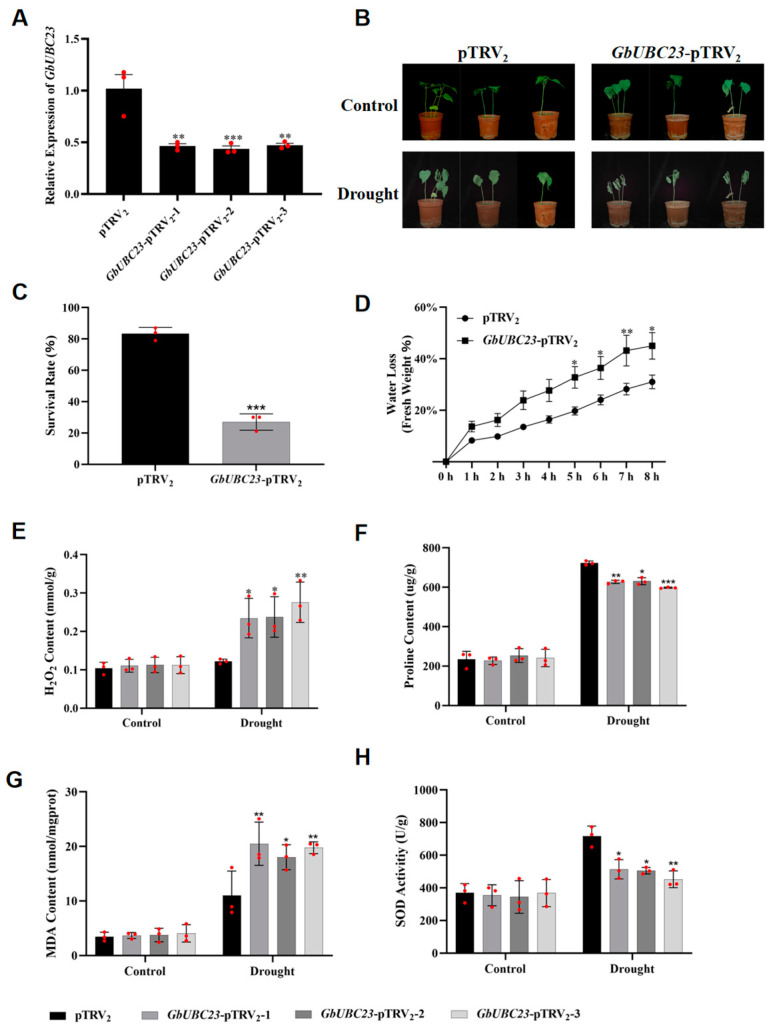
Silencing of the *GbUBC23* gene diminishes the drought tolerance of sea-island cotton. (**A**) Relative expression levels of *GbUBC23* in pTRV2 and GbUBC23-pTRV2 cotton; (**B**) identification of drought-resistant phenotypes in sea-island cotton; (**C**) statistics on survival rates under drought conditions; (**D**) rate of water loss in detached leaves; (**E**) quantity of H_2_O_2_ prior to and after drought stress; (**F**) MDA contents before and after drought stress; (**G**) proline content levels prior to and after drought stress; (**H**) SOD contents before and after drought stress. The vertical bars denote the averages ± standard deviations, with significant differences from control plants shown by * *p* < 0.01, ** *p* < 0.001, and *** *p* < 0.0001.

To understand the drought resistance effect of *GbUBC23*, we silenced the *GbUBC23* gene in sea-island cotton. Following the injection of the positive control gene *GbCLA1* into sea-island cotton seedlings, the leaves of the seedlings were bleached after seven days (Supplementary Figure S1), and this state persisted for more than one month. This finding indicates potentially successful gene silencing. Afterward, qPCR was used to measure *GbUBC23* gene expression in the control and *GbUBC23*-silenced plants. Compared with that in the control plants, the expression level of the *GbUBC23* gene in the silenced plants was much lower, indicating that the *GbUBC23* gene was successfully silenced in cotton (Figure 9A).

Before drought stress, no notable phenotypic abnormalities were observed between the control and silenced plants; however, after drought stress, the severity of wilting in the silenced plants was significantly greater than that in the control plants (Figure 9B). The survival rate of the control plants was 83.3%, whereas the survival rate of the silenced plants was 27%, representing a substantial decrease (Figure 9C). Notably, over a timeframe ranging from five to eight hours, the rate of water loss from the detached leaves of the silenced plants was much greater than that of the control plants (Figure 9D). Under abiotic stress, plants accumulate reactive oxygen species (ROS) in their tissues, accompanied by increases in the levels of H_2_O_2_ and MDA. Before drought treatment, the H_2_O_2_ and MDA levels in the leaves of the silenced plants were the same as those in the control plants. After drought treatment, the H_2_O_2_ and MDA levels were considerably greater in the leaves of the silenced plants than in those of the control plants (Figure 9E,G). As an osmotic regulator, proline maintains the stability of plant cells under drought stress. The leaves of the silenced plants had the same levels of proline as the controls before drought treatment. Compared with those in the control plants, the proline concentrations in the leaves of the silenced plants were markedly lower after drought treatment, indicating that the silencing of *GbUBC23* impaired proline synthesis under drought conditions (Figure 9F). Additionally, we assessed the activity of the antioxidant enzyme SOD in both the silenced and control plants. During drought stress, the activity of SOD in the silenced plants was markedly lower than that in the control plants; however, prior to drought stress, no significant difference in SOD activity was noted between the two groups (Figure 9H). Taken together, these results demonstrate that *GbUBC23* exerts a beneficial regulatory effect on sea-island cotton under drought stress.

### 2.9. Changes in Drought Stress-Related Gene Expression

**Figure 10:**
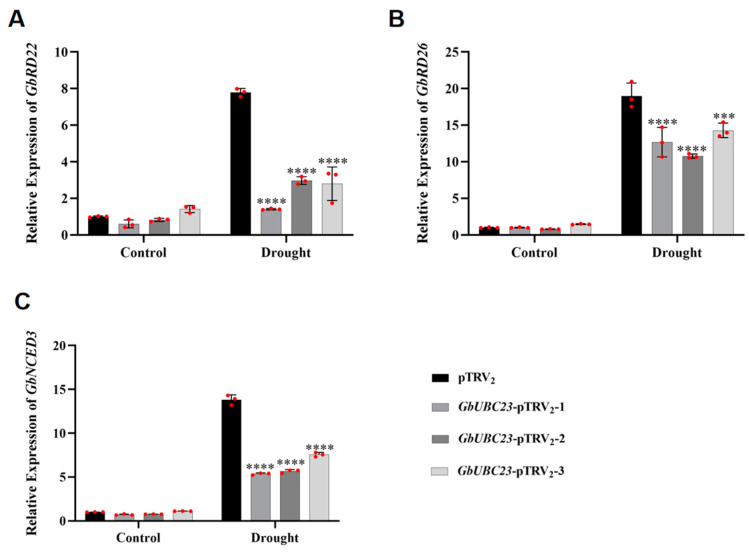
Expression levels of drought-responsive genes in GbUBC23-silenced cotton. (**A**) *GbRD22* (*Gbar_A05G004580.4*) expression; (**B**) *GbRD26* expression (*Gbar_A01G005760.1*); (**C**) *GbNCED3* (*Gbar_A13G016670.1*) expression. The vertical bars denote the averages ± standard deviations, with significant changes relative to pTRV2 indicated by *** *p* < 0.0001 and **** *p* < 0.0001.

Silencing of the *GbUBC23* gene resulted in a reduction in the drought resistance of sea-island cotton. As a result, changes in the expression of genes associated with drought stress were observed to elucidate the molecular mechanism of *GbUBC23*. The expression levels of the genes *GbNCED3*, *GbRD22*, and *GbRD26*, which are associated with drought stress, were analyzed. Under normal circumstances, the expression levels of *GbNCED3*, *GbRD22*, and *GbRD26* were not significantly different between the silenced and control plants (Figure 10). Under drought stress conditions, the expression levels of three drought stress-related genes in both the silenced and control plants markedly increased compared with the prestress values. Nonetheless, the expression levels in the silenced plants were markedly lower than those recorded in the control plants (Figure 10). These findings suggest that the repression of the *GbUBC23* gene significantly influences cotton drought tolerance.

## 3. Discussion

Protein ubiquitination is a key process in maintaining cellular homeostasis. E2s are key enzymes involved in protein ubiquitination, providing a binding platform for the ubiquitination of E1s and E3s [33]. Therefore, E2s play crucial roles in normal cellular function. Recently, numerous E2s have been identified in plants, including 48 in rice [34], 75 in maize [20], 53 in *Arabidopsis* [5,8,35], and 169 in upland cotton [9]. This study identified 125 members of the *GbUBC* gene family, a greater number than the 53 *AtUBC* genes but fewer than the 169 *GhUBC* genes. A total of 347 UBC proteins from *Arabidopsis*, upland cotton, and sea-island cotton were then grouped into 21 subgroups with tight genetic links via phylogenetic tree analysis [9]. Subcellular localization prediction revealed that most GbUBCs reside in the nucleus, whereas a minority reside in other organelles, suggesting that these proteins regulate microenvironments. According to conserved motif analysis, each member contains at least one UBC-conserved domain with high consistency. An analysis of the gene structure revealed that most members contained 5–6 exons, but there were significant differences in length and structure, indicating that the gene structure of the *GbUBC* genes is relatively complex. A predictive study of *GbUBC* gene promoters revealed that most genes have growth-, development-, and abiotic stress-responsive *cis*-acting elements. The findings of this study indicate that genes belonging to the GbUBC family may be involved in the regulation of growth, development, and response to abiotic stress in sea-island cotton. Gene duplication is an important mechanism in the evolution of gene families. Repeated gene duplication occurs in plants, leading to gene combination or amplification, thereby expanding the gene family. According to previous reports, gene duplication has also occurred in members of the UBC family in wheat [10], rice [14], maize [20], and potato [16]. Through collinearity analysis, we found that 79.2% of the *GbUBC* genes had undergone duplication events and that 98 pairs of duplicated genes had undergone segmental duplication. The majority of the Ka/Ks values were less than 1, suggesting that the *GbUBC* genes have been conserved during sea-island cotton genome evolution via segmental duplication, thereby contributing to the expansion of the *GbUBC* gene family in this species.

Our analysis of *GbUBC* gene family gene expression levels in sea-island cotton indicates that genes within the same subgroup typically exhibit analogous expression patterns across various tissues, developmental stages, and abiotic stress conditions. Almost all the *GbUBC* family genes were highly expressed under different abiotic stresses, indicating their potential role in the response of sea-island cotton to these stresses. Studying the spatiotemporal expression pattern of the *GbUBC* gene provides valuable information for understanding its potential function in sea-island cotton. Previous research has indicated that the expression of numerous UBC genes across various plant species is affected by tissue type, developmental stage, and environmental conditions [9,36,37].

Previous studies revealed a high degree of similarity (71%) between ZmUBC55 and GbUBC23, and *ZmUBC55* was upregulated under PEG stress [20]; in addition, GbTCP5 activated the expression of *GbUBC23* through the TCP *cis*-acting element in the promoter region of the *GbUBC23* gene, regulating drought tolerance in sea-island cotton [38]. Therefore, the *GbUBC23* gene is likely involved in drought stress in sea-island cotton. We subsequently identified *GbUBC23*, and the expression data revealed that *GbUBC23* was highly expressed in cotyledons and highly expressed after 24 h of PEG-simulated drought stress, indicating that *GbUBC23* is involved in the regulation of plant responses to abiotic stress. GbUBC23 is highly similar to UBC proteins in the same subfamily of *Arabidopsis* and upland cotton, and both have a conserved cysteine residue, indicating that GbUBC23 also functions through classical ubiquitination [39]. Subcellular localization can reveal which organelle proteins act on, and many plant UBC proteins localize to multiple organelles [39]. Similarly, GbUBC23 is also localized in the nucleus and cell membrane [19,40].

ROS in plants are essential for sensing biotic and abiotic stress, integrating various environmental signals, and activating stress-response networks [41,42]. An increase in H_2_O_2_ content in upland cotton and tomato reduces the drought tolerance of these plants [43,44]. Many plants experience an increase in MDA due to drought stress [45,46]. Under drought stress, plants increase their drought tolerance by reducing their ROS content via increased SOD activity [47,48]. TaERF87 and TaAKS1 work together to control the production of proline mediated by TaP5CS1/TaP5CR1 to promote drought resistance in wheat [49]. In addition, overexpression of the *GmUBC9* gene in soybean can increase the proline content and reduce the MDA content, thereby increasing soybean drought resistance [19]. The overexpression of *AhUBC2* in *Arabidopsis* can increase the proline content and increase drought tolerance [50]. In our research, we found that silencing the *GbUBC23* gene in sea-island cotton before drought did not result in significant differences in phenotype between the silenced *GbUBC23* plants and the control plants. Physiological indicators related to drought stress, such as H_2_O_2_ content, MDA content, proline content, and SOD activity, also did not significantly differ. Compared with those of the control plants, the detached leaves of the silenced *GbUBC23* plants lost water at a substantially faster rate. However, compared with the control plants, the plants in which *GbUBC23* was silenced after drought stress were more prone to wilting, presented increased levels of H_2_O_2_ and MDA, and presented reduced concentrations of proline and SOD. These results demonstrate that the silencing of *GbUBC23* diminishes drought tolerance in sea-island cotton. One of the drought resistance mechanisms of *GbUBC23* involves alleviating the accumulation of ROS under drought stress.

Previous studies identified *RD22*, *RD26*, and *NCED3* as marker genes for the abiotic stress response in *Arabidopsis*. The *RD22* gene is upregulated in response to drought and abscisic acid [49]. RD26 is an NAC transcription factor that is activated by drought and abscisic acid stimulation [51]. By synthesizing abscisic acid, *NCED3* increases drought tolerance in *Arabidopsis* [52]. Our study revealed the expression levels of the *GbRD22*, *GbRD26*, and *GbNCED3* genes, which are homologous to those in *Arabidopsis* in sea-island cotton. Before drought stress, the expression levels in the control plants and those in the silenced plants did not differ significantly. Compared with the control plants, the silenced plants presented significantly lower expression levels of *GbRD22*, *GbRD26*, and *GbNCED3*. These findings indicate that *GbUBC23* is involved in the activation of stress-responsive gene expression, ultimately reducing the drought tolerance of transgenic sea-island cotton.

## 4. Materials and Methods

### 4.1. Identification of the GbUBC Gene Family in Sea-Island Cotton

The complete genome of sea-island cotton was downloaded from a database (https://yanglab.hzau.edu.cn/CottonMD.1) (accessed on 5 November 2024). Additionally, Pfam (http://pfam.xfam.org) (accessed on 5 November 2024) was employed to obtain the hidden Markov model (HMM) profile for the UBC domain (PF00179) [53], and the whole-genome protein sequence of sea-island cotton was examined via HMMER 3.0 [54]. The NCBI Conserved Domain Database (NCBI-CDD) (https://www.ncbi.nlm.nih.gov/Structure/bwrpsb/bwrpsb.cgi) (accessed on 5 November 2024), InterPro (https://www.ebi.ac.uk/interpro/) (accessed on 5 November 2024), and SMART (https://smart.embl.de/) (accessed on 5 November 2024) were utilized to verify that the GbUBC proteins possessed structural domain characteristics of E2 ubiquitin-conjugating enzymes [55,56,57]. Genes lacking UBC domains and those that were not full-length were manually eliminated. The average hydropathicity, aliphatic index, pI, and MW of each GbUBC amino acid sequence were concurrently predicted via the web tool ExPASy-PROSITE (http://www.expasy.org/) (accessed on 10 November 2024) [58]. Plant-mPLoc (http://www.csbio.sjtu.edu.cn/bioinf/plant-multi/) (accessed on 10 November 2024) was employed for subcellular localization prediction [59].

### 4.2. Sequence Alignment and Phylogenetic Analysis

The Arabidopsis Information Resource (TAIR) database (http://Arabidopsis.org) (accessed on 10 November 2024) was utilized to obtain the 53 AtUBC protein sequences. The 169 GhUBC protein sequences were downloaded from the cotton database (https://yanglab.hzau.edu.cn/CottonMD.1) (accessed on 10 November 2024) (Table S1). MEGA6 software was subsequently used for multiple sequence alignment via the neighbor-joining (NJ) technique with 1000 iterations, and a phylogenetic tree was generated by integrating the GbUBC protein sequence with the UBC protein sequences from *Arabidopsis* and upland cotton. The ITOL online application (https://itol.embl.de/) (accessed on 10 November 2024) was utilized to display the phylogenetic tree [60].

### 4.3. Gene Structure and Protein-Conserved Motifs

The length and distribution statistics of the matching introns and exons were retrieved from the cotton database in accordance with the cDNA sequence of the UBC genes of sea-island cotton, the gene structure of the *GbUBC* gene family was analyzed via TBtools v2.136 software [61], and the protein-conserved motifs of the UBC family of sea-island cotton were predicted via the MEME website (http://meme-suite.org/index.html) (accessed on 12 November 2024). The data were ultimately visualized via TBtools software.

### 4.4. Analysis of the Cis-Regulatory Elements in the Promoter

TBtools software was used to obtain 2.0-kb DNA sequences upstream from the initiation codon of the *GbUBC* gene, and the promoter *cis*-acting elements of UBC family members of sea-island cotton were predicted via PlantCARE. Ultimately, TBtools software was used for data visualization.

### 4.5. Chromosomal Location, Gene Duplication, and Collinearity Analysis

Using TBtools, the *GbUBC* genes were mapped to chromosomes from the cotton database (https://yanglab.hzau.edu.cn/CottonMD.1) (accessed on 12 November 2024). For pairs of duplicate genes, the simple Ka/Ks calculation module calculates synonymous and nonsynonymous substitution rates. A Ka/Ks value greater than 1 indicates positive selection, a value equal to 1 represents neutral selection, and a value less than 1 indicates purifying selection [62].

### 4.6. GbUBC Gene Expression Profiles

The cotton expression database (https://yanglab.hzau.edu.cn/CottonMD/expression.1) (accessed on 12 November 2024) provides RNA-seq data from various tissues and organs, as well as from plants exposed to a range of abiotic stress treatments and developmental phases. An internet platform (https://www.bioinformatics.com.cn) (accessed on 13 November 2024) was used to generate heatmaps [63].

### 4.7. RNA Extraction and qPCR Analysis

Total RNA was extracted via a plant total RNA extraction kit (Tiangen Biochemical Technology (Beijing) Co., Ltd., Beijing, China). Following the manufacturer’s instructions (Tiangen Biochemical Technology (Beijing) Co., Ltd., Beijing, China), EasyScript One-Step gDNA Removal and cDNA Synthesis SuperMix EasyScript were used to synthesize first-strand cDNA. Reagents such as SYBR and MixTaq (TransGen Biotech (Beijing) Co., Ltd., Beijing, China) were utilized, and quantitative polymerase chain reaction (qPCR) was carried out via an ABI 7500 apparatus, with a total volume of 20 μL. Cotton *GbUBQ7* served as an internal reference gene [38]. The 2^−ΔΔct^ technique was used to determine the relative amount, and the data were processed via Excel 2019 software [64]. The primers used for qPCR are detailed in Table S4.

### 4.8. Subcellular Localization

We used a vector that encodes GFP and added a stop codon-free GbUBC23 coding sequence near the N-terminal region of GFP to construct 35S::GbUBC23-GFP for subcellular localization of GbUBC23. The engineered vectors were subsequently transformed into *Agrobacterium* GV3101, which was subsequently introduced into the leaves of 4-week-old *Nicotiana benthamiana* plants. Next, the inner epidermis of the onion was submerged in the aforementioned *Agrobacterium* and cultivated in sterile MS media. Observations were made after a period of 3 days via a confocal laser microscope (Zeiss LSM 800, Carl Zeiss, Germany). ZEN Imaging Software Version 3.4 was used to collect the fluorescence signal carefully and produce high-quality photographs. Table S4 presents a summary of the specific sequences of the primers used.

### 4.9. Virus-Induced Gene Silencing (VIGS) of GbUBC23

We used pTRV2 as the virus-silencing vector. We ligated the 320 bp untranslated region of *GbUBC23* to the pTRV2 vector through homologous recombination, resulting in GbUBC23-pTRV2. We subsequently introduced the virus into *Agrobacterium* GV3101 via the freeze-thaw technique. The cotton injection process was conducted following Zhang’s guidelines [65], utilizing sea-island cotton samples that had been infused with the *GbCLA1* gene as a positive control. qPCR was used to assess the silencing effect. All trials were performed at least three times, with the experimental design guaranteeing that each duplicate included a minimum of 30 sea-island cotton plants. The primers used for PCR and qPCR are listed in Table S4.

### 4.10. Plant Materials and Treatments

XH61 sea-island cotton was used as the receptor for VIGS transient transformation and for qPCR experiments. The plant materials used for subcellular localization were *N. benthamiana* and onion. The seeds of the sea-island cotton variety XH61 were planted directly in black soil. The ratio of black soil to vermiculite was 2:1. The soil was maintained at a temperature of 28 °C, and the cycle consisted of 16 h of light and 8 h of darkness. The robust three-leaf sea-island cotton was transferred to hydroponic cultivation for treatment. After a two-day recovery period, the cotton roots were submerged in a 20% PEG6000 solution supplemented with Hoagland nutrition solution.

### 4.11. Determination of Physiological and Biochemical Indices

To determine the survival rate of the silenced plants, the control and silenced plants were grown under the same conditions for 7 days, after which the water was stopped until the plants exhibited an obvious wilting phenotype. The plants with a wilting phenotype were counted. For the determination of water loss in sea-island cotton leaves, the leaves of the control and those of the silenced plants were promptly detached and weighed on weighing paper. The samples were subsequently positioned on filter paper, and the variation in fresh weight over time was documented at 25 °C and 70% relative humidity. The fresh weight loss of the sample was used to determine the water-loss rate of the isolated leaves. The H_2_O_2_ content, MDA content, proline content, and SOD content were determined via a Nanjing Jiangcheng kit.

### 4.12. Statistical Analysis

GraphPad Prism 9.0 was used to chart the data, and analysis of variance (ANOVA) and Student’s *t* test were used for data analysis.

## 5. Conclusions

In summary, we analyzed 125 *GbUBC* genes in sea-island cotton and determined that *GbUBC23* can increase the drought resistance of sea-island cotton. Although the mechanism by which *GbUBC23* responds to drought is not fully understood, *GbUBC23* may be involved in the abscisic acid pathway and the clearance of ROS to increase drought resistance. This study not only elucidates the role of *GbUBC23* under drought stress but also provides useful tools for crop molecular breeding and biotechnology development.

## Data Availability

All data generated during this study are included within the article or its Supplementary Files.

## Supplementary Materials

The following supporting information can be downloaded at: https://www.mdpi.com/article/10.3390/ijms252312948/s1.
