# Supplementary material for: Genome-Wide Identification of the GbUBC Gene Family in Sea-Island Cotton (Gossypium barbadense) and the Active Regulation of Drought Resistance in Cotton by GbUBC23
Source: Int J Mol Sci. 2024 Dec 2;25(23):12948. doi: 10.3390/ijms252312948 (PMC11640981; doi:10.3390/ijms252312948)
Supplement: Supplementary file 1 [file ijms-25-12948-s001.zip › Table S1.pdf]

Table S1 The database and accession number of the genetic information of the plants

Genetic Information Database	Version Number	Species	Gene ID	Gene Name
https://yanglab.hzau.edu.cn/CottonMD/download.1	3-79_HAU	*Gossypium barbadense*	Gbar_A01G001550.1	GbUBC1
https://yanglab.hzau.edu.cn/CottonMD/download.1	3-79_HAU	*Gossypium barbadense*	Gbar_A01G004680.1	GbUBC2
https://yanglab.hzau.edu.cn/CottonMD/download.1	3-79_HAU	*Gossypium barbadense*	Gbar_A01G005430.4	GbUBC3
https://yanglab.hzau.edu.cn/CottonMD/download.1	3-79_HAU	*Gossypium barbadense*	Gbar_A01G010590.1	GbUBC4
https://yanglab.hzau.edu.cn/CottonMD/download.1	3-79_HAU	*Gossypium barbadense*	Gbar_A01G014530.1	GbUBC5
https://yanglab.hzau.edu.cn/CottonMD/download.1	3-79_HAU	*Gossypium barbadense*	Gbar_A01G016510.1	GbUBC6
https://yanglab.hzau.edu.cn/CottonMD/download.1	3-79_HAU	*Gossypium barbadense*	Gbar_A01G020060.3	GbUBC7
https://yanglab.hzau.edu.cn/CottonMD/download.1	3-79_HAU	*Gossypium barbadense*	Gbar_A02G000160.1	GbUBC8
https://yanglab.hzau.edu.cn/CottonMD/download.1	3-79_HAU	*Gossypium barbadense*	Gbar_A02G010830.1	GbUBC9
https://yanglab.hzau.edu.cn/CottonMD/download.1	3-79_HAU	*Gossypium barbadense*	Gbar_A02G018000.1	GbUBC10
https://yanglab.hzau.edu.cn/CottonMD/download.1	3-79_HAU	*Gossypium barbadense*	Gbar_A03G002590.2	GbUBC11
https://yanglab.hzau.edu.cn/CottonMD/download.1	3-79_HAU	*Gossypium barbadense*	Gbar_A03G004830.1	GbUBC12
https://yanglab.hzau.edu.cn/CottonMD/download.1	3-79_HAU	*Gossypium barbadense*	Gbar_A03G013550.3	GbUBC13
https://yanglab.hzau.edu.cn/CottonMD/download.1	3-79_HAU	*Gossypium barbadense*	Gbar_A03G017430.3	GbUBC14
https://yanglab.hzau.edu.cn/CottonMD/download.1	3-79_HAU	*Gossypium barbadense*	Gbar_A04G000140.1	GbUBC15
https://yanglab.hzau.edu.cn/CottonMD/download.1	3-79_HAU	*Gossypium barbadense*	Gbar_A04G003790.1	GbUBC16
https://yanglab.hzau.edu.cn/CottonMD/download.1	3-79_HAU	*Gossypium barbadense*	Gbar_A04G013530.1	GbUBC17
https://yanglab.hzau.edu.cn/CottonMD/download.1	3-79_HAU	*Gossypium barbadense*	Gbar_A04G015330.1	GbUBC18
https://yanglab.hzau.edu.cn/CottonMD/download.1	3-79_HAU	*Gossypium barbadense*	Gbar_A05G009350.1	GbUBC19
https://yanglab.hzau.edu.cn/CottonMD/download.1	3-79_HAU	*Gossypium barbadense*	Gbar_A05G018740.1	GbUBC20
https://yanglab.hzau.edu.cn/CottonMD/download.1	3-79_HAU	*Gossypium barbadense*	Gbar_A05G020330.3	GbUBC21
https://yanglab.hzau.edu.cn/CottonMD/download.1	3-79_HAU	*Gossypium barbadense*	Gbar_A05G027100.1	GbUBC22
https://yanglab.hzau.edu.cn/CottonMD/download.1	3-79_HAU	*Gossypium barbadense*	Gbar_A05G029480.1	GbUBC23
https://yanglab.hzau.edu.cn/CottonMD/download.1	3-79_HAU	*Gossypium barbadense*	Gbar_A05G041600.1	GbUBC24
https://yanglab.hzau.edu.cn/CottonMD/download.1	3-79_HAU	*Gossypium barbadense*	Gbar_A06G006360.2	GbUBC25
https://yanglab.hzau.edu.cn/CottonMD/download.1	3-79_HAU	*Gossypium barbadense*	Gbar_A06G009810.1	GbUBC26
https://yanglab.hzau.edu.cn/CottonMD/download.1	3-79_HAU	*Gossypium barbadense*	Gbar_A06G010890.2	GbUBC27
https://yanglab.hzau.edu.cn/CottonMD/download.1	3-79_HAU	*Gossypium barbadense*	Gbar_A06G013380.1	GbUBC28
https://yanglab.hzau.edu.cn/CottonMD/download.1	3-79_HAU	*Gossypium barbadense*	Gbar_A06G015420.1	GbUBC29
https://yanglab.hzau.edu.cn/CottonMD/download.1	3-79_HAU	*Gossypium barbadense*	Gbar_A07G002770.1	GbUBC30
https://yanglab.hzau.edu.cn/CottonMD/download.1	3-79_HAU	*Gossypium barbadense*	Gbar_A07G006630.1	GbUBC31
https://yanglab.hzau.edu.cn/CottonMD/download.1	3-79_HAU	*Gossypium barbadense*	Gbar_A07G010190.1	GbUBC32
https://yanglab.hzau.edu.cn/CottonMD/download.1	3-79_HAU	*Gossypium barbadense*	Gbar_A07G012900.1	GbUBC33
https://yanglab.hzau.edu.cn/CottonMD/download.1	3-79_HAU	*Gossypium barbadense*	Gbar_A08G003010.1	GbUBC34
https://yanglab.hzau.edu.cn/CottonMD/download.1	3-79_HAU	*Gossypium barbadense*	Gbar_A08G011230.1	GbUBC35
https://yanglab.hzau.edu.cn/CottonMD/download.1	3-79_HAU	*Gossypium barbadense*	Gbar_A08G016540.1	GbUBC36
https://yanglab.hzau.edu.cn/CottonMD/download.1	3-79_HAU	*Gossypium barbadense*	Gbar_A08G023610.1	GbUBC37
https://yanglab.hzau.edu.cn/CottonMD/download.1	3-79_HAU	*Gossypium barbadense*	Gbar_A09G012500.1	GbUBC38
https://yanglab.hzau.edu.cn/CottonMD/download.1	3-79_HAU	*Gossypium barbadense*	Gbar_A09G018180.1	GbUBC39
https://yanglab.hzau.edu.cn/CottonMD/download.1	3-79_HAU	*Gossypium barbadense*	Gbar_A09G019350.1	GbUBC40
https://yanglab.hzau.edu.cn/CottonMD/download.1	3-79_HAU	*Gossypium barbadense*	Gbar_A09G022840.1	GbUBC41

https://yanglab.hzau.edu.cn/CottonMD/download.1	3-79_HAU	*Gossypium barbadense*	Gbar_A09G025070.1	GbUBC42
https://yanglab.hzau.edu.cn/CottonMD/download.1	3-79_HAU	*Gossypium barbadense*	Gbar_A09G025130.1	GbUBC43
https://yanglab.hzau.edu.cn/CottonMD/download.1	3-79_HAU	*Gossypium barbadense*	Gbar_A10G002820.1	GbUBC44
https://yanglab.hzau.edu.cn/CottonMD/download.1	3-79_HAU	*Gossypium barbadense*	Gbar_A10G005430.1	GbUBC45
https://yanglab.hzau.edu.cn/CottonMD/download.1	3-79_HAU	*Gossypium barbadense*	Gbar_A10G016430.1	GbUBC46
https://yanglab.hzau.edu.cn/CottonMD/download.1	3-79_HAU	*Gossypium barbadense*	Gbar_A10G018400.1	GbUBC47
https://yanglab.hzau.edu.cn/CottonMD/download.1	3-79_HAU	*Gossypium barbadense*	Gbar_A11G000970.1	GbUBC48
https://yanglab.hzau.edu.cn/CottonMD/download.1	3-79_HAU	*Gossypium barbadense*	Gbar_A11G001850.1	GbUBC49
https://yanglab.hzau.edu.cn/CottonMD/download.1	3-79_HAU	*Gossypium barbadense*	Gbar_A11G001980.1	GbUBC50
https://yanglab.hzau.edu.cn/CottonMD/download.1	3-79_HAU	*Gossypium barbadense*	Gbar_A11G003890.1	GbUBC51
https://yanglab.hzau.edu.cn/CottonMD/download.1	3-79_HAU	*Gossypium barbadense*	Gbar_A11G004570.1	GbUBC52
https://yanglab.hzau.edu.cn/CottonMD/download.1	3-79_HAU	*Gossypium barbadense*	Gbar_A11G009790.1	GbUBC53
https://yanglab.hzau.edu.cn/CottonMD/download.1	3-79_HAU	*Gossypium barbadense*	Gbar_A11G017860.1	GbUBC54
https://yanglab.hzau.edu.cn/CottonMD/download.1	3-79_HAU	*Gossypium barbadense*	Gbar_A11G019160.1	GbUBC55
https://yanglab.hzau.edu.cn/CottonMD/download.1	3-79_HAU	*Gossypium barbadense*	Gbar_A11G020100.2	GbUBC56
https://yanglab.hzau.edu.cn/CottonMD/download.1	3-79_HAU	*Gossypium barbadense*	Gbar_A11G024410.3	GbUBC57
https://yanglab.hzau.edu.cn/CottonMD/download.1	3-79_HAU	*Gossypium barbadense*	Gbar_A11G030160.1	GbUBC58
https://yanglab.hzau.edu.cn/CottonMD/download.1	3-79_HAU	*Gossypium barbadense*	Gbar_A11G030910.2	GbUBC59
https://yanglab.hzau.edu.cn/CottonMD/download.1	3-79_HAU	*Gossypium barbadense*	Gbar_A11G032790.1	GbUBC60
https://yanglab.hzau.edu.cn/CottonMD/download.1	3-79_HAU	*Gossypium barbadense*	Gbar_A11G033860.1	GbUBC61
https://yanglab.hzau.edu.cn/CottonMD/download.1	3-79_HAU	*Gossypium barbadense*	Gbar_A11G033870.1	GbUBC62
https://yanglab.hzau.edu.cn/CottonMD/download.1	3-79_HAU	*Gossypium barbadense*	Gbar_A11G033880.1	GbUBC63
https://yanglab.hzau.edu.cn/CottonMD/download.1	3-79_HAU	*Gossypium barbadense*	Gbar_A12G003670.1	GbUBC64
https://yanglab.hzau.edu.cn/CottonMD/download.1	3-79_HAU	*Gossypium barbadense*	Gbar_A12G006330.1	GbUBC65
https://yanglab.hzau.edu.cn/CottonMD/download.1	3-79_HAU	*Gossypium barbadense*	Gbar_A12G006800.1	GbUBC66
https://yanglab.hzau.edu.cn/CottonMD/download.1	3-79_HAU	*Gossypium barbadense*	Gbar_A12G017830.1	GbUBC67
https://yanglab.hzau.edu.cn/CottonMD/download.1	3-79_HAU	*Gossypium barbadense*	Gbar_A12G018720.2	GbUBC68
https://yanglab.hzau.edu.cn/CottonMD/download.1	3-79_HAU	*Gossypium barbadense*	Gbar_A12G020550.1	GbUBC69
https://yanglab.hzau.edu.cn/CottonMD/download.1	3-79_HAU	*Gossypium barbadense*	Gbar_A12G026340.1	GbUBC70
https://yanglab.hzau.edu.cn/CottonMD/download.1	3-79_HAU	*Gossypium barbadense*	Gbar_A12G029100.1	GbUBC71
https://yanglab.hzau.edu.cn/CottonMD/download.1	3-79_HAU	*Gossypium barbadense*	Gbar_A13G024300.1	GbUBC72
https://yanglab.hzau.edu.cn/CottonMD/download.1	3-79_HAU	*Gossypium barbadense*	Gbar_D01G001720.1	GbUBC73
https://yanglab.hzau.edu.cn/CottonMD/download.1	3-79_HAU	*Gossypium barbadense*	Gbar_D01G003810.1	GbUBC74
https://yanglab.hzau.edu.cn/CottonMD/download.1	3-79_HAU	*Gossypium barbadense*	Gbar_D01G005660.5	GbUBC75
https://yanglab.hzau.edu.cn/CottonMD/download.1	3-79_HAU	*Gossypium barbadense*	Gbar_D01G011150.1	GbUBC76
https://yanglab.hzau.edu.cn/CottonMD/download.1	3-79_HAU	*Gossypium barbadense*	Gbar_D01G015390.1	GbUBC77
https://yanglab.hzau.edu.cn/CottonMD/download.1	3-79_HAU	*Gossypium barbadense*	Gbar_D01G021190.1	GbUBC78
https://yanglab.hzau.edu.cn/CottonMD/download.1	3-79_HAU	*Gossypium barbadense*	Gbar_D02G000270.1	GbUBC79
https://yanglab.hzau.edu.cn/CottonMD/download.1	3-79_HAU	*Gossypium barbadense*	Gbar_D02G015200.1	GbUBC80
https://yanglab.hzau.edu.cn/CottonMD/download.1	3-79_HAU	*Gossypium barbadense*	Gbar_D02G019250.6	GbUBC81
https://yanglab.hzau.edu.cn/CottonMD/download.1	3-79_HAU	*Gossypium barbadense*	Gbar_D03G015870.2	GbUBC82
https://yanglab.hzau.edu.cn/CottonMD/download.1	3-79_HAU	*Gossypium barbadense*	Gbar_D04G000310.1	GbUBC83
https://yanglab.hzau.edu.cn/CottonMD/download.1	3-79_HAU	*Gossypium barbadense*	Gbar_D04G018200.1	GbUBC84

https://yanglab.hzau.edu.cn/CottonMD/download.1	3-79_HAU	*Gossypium barbadense*	Gbar_D04G020140.1	GbUBC85
https://yanglab.hzau.edu.cn/CottonMD/download.1	3-79_HAU	*Gossypium barbadense*	Gbar_D05G009810.1	GbUBC86
https://yanglab.hzau.edu.cn/CottonMD/download.1	3-79_HAU	*Gossypium barbadense*	Gbar_D05G019440.1	GbUBC87
https://yanglab.hzau.edu.cn/CottonMD/download.1	3-79_HAU	*Gossypium barbadense*	Gbar_D05G021000.1	GbUBC88
https://yanglab.hzau.edu.cn/CottonMD/download.1	3-79_HAU	*Gossypium barbadense*	Gbar_D05G027950.1	GbUBC89
https://yanglab.hzau.edu.cn/CottonMD/download.1	3-79_HAU	*Gossypium barbadense*	Gbar_D05G030260.1	GbUBC90
https://yanglab.hzau.edu.cn/CottonMD/download.1	3-79_HAU	*Gossypium barbadense*	Gbar_D05G035090.2	GbUBC91
https://yanglab.hzau.edu.cn/CottonMD/download.1	3-79_HAU	*Gossypium barbadense*	Gbar_D06G006640.1	GbUBC92
https://yanglab.hzau.edu.cn/CottonMD/download.1	3-79_HAU	*Gossypium barbadense*	Gbar_D06G010300.1	GbUBC93
https://yanglab.hzau.edu.cn/CottonMD/download.1	3-79_HAU	*Gossypium barbadense*	Gbar_D06G012060.1	GbUBC94
https://yanglab.hzau.edu.cn/CottonMD/download.1	3-79_HAU	*Gossypium barbadense*	Gbar_D06G014120.1	GbUBC95
https://yanglab.hzau.edu.cn/CottonMD/download.1	3-79_HAU	*Gossypium barbadense*	Gbar_D06G016040.1	GbUBC96
https://yanglab.hzau.edu.cn/CottonMD/download.1	3-79_HAU	*Gossypium barbadense*	Gbar_D07G006930.1	GbUBC97
https://yanglab.hzau.edu.cn/CottonMD/download.1	3-79_HAU	*Gossypium barbadense*	Gbar_D07G010570.2	GbUBC98
https://yanglab.hzau.edu.cn/CottonMD/download.1	3-79_HAU	*Gossypium barbadense*	Gbar_D07G013300.1	GbUBC99
https://yanglab.hzau.edu.cn/CottonMD/download.1	3-79_HAU	*Gossypium barbadense*	Gbar_D08G003120.1	GbUBC100
https://yanglab.hzau.edu.cn/CottonMD/download.1	3-79_HAU	*Gossypium barbadense*	Gbar_D08G017260.1	GbUBC101
https://yanglab.hzau.edu.cn/CottonMD/download.1	3-79_HAU	*Gossypium barbadense*	Gbar_D09G017910.1	GbUBC102
https://yanglab.hzau.edu.cn/CottonMD/download.1	3-79_HAU	*Gossypium barbadense*	Gbar_D09G019160.1	GbUBC103
https://yanglab.hzau.edu.cn/CottonMD/download.1	3-79_HAU	*Gossypium barbadense*	Gbar_D09G024720.1	GbUBC104
https://yanglab.hzau.edu.cn/CottonMD/download.1	3-79_HAU	*Gossypium barbadense*	Gbar_D09G024780.1	GbUBC105
https://yanglab.hzau.edu.cn/CottonMD/download.1	3-79_HAU	*Gossypium barbadense*	Gbar_D10G002720.1	GbUBC106
https://yanglab.hzau.edu.cn/CottonMD/download.1	3-79_HAU	*Gossypium barbadense*	Gbar_D10G005260.1	GbUBC107
https://yanglab.hzau.edu.cn/CottonMD/download.1	3-79_HAU	*Gossypium barbadense*	Gbar_D10G011720.1	GbUBC108
https://yanglab.hzau.edu.cn/CottonMD/download.1	3-79_HAU	*Gossypium barbadense*	Gbar_D10G018560.1	GbUBC109
https://yanglab.hzau.edu.cn/CottonMD/download.1	3-79_HAU	*Gossypium barbadense*	Gbar_D11G001330.1	GbUBC110
https://yanglab.hzau.edu.cn/CottonMD/download.1	3-79_HAU	*Gossypium barbadense*	Gbar_D11G002190.1	GbUBC111
https://yanglab.hzau.edu.cn/CottonMD/download.1	3-79_HAU	*Gossypium barbadense*	Gbar_D11G010330.1	GbUBC112
https://yanglab.hzau.edu.cn/CottonMD/download.1	3-79_HAU	*Gossypium barbadense*	Gbar_D11G018780.1	GbUBC113
https://yanglab.hzau.edu.cn/CottonMD/download.1	3-79_HAU	*Gossypium barbadense*	Gbar_D11G031220.1	GbUBC114
https://yanglab.hzau.edu.cn/CottonMD/download.1	3-79_HAU	*Gossypium barbadense*	Gbar_D11G033560.1	GbUBC115
https://yanglab.hzau.edu.cn/CottonMD/download.1	3-79_HAU	*Gossypium barbadense*	Gbar_D11G036910.1	GbUBC116
https://yanglab.hzau.edu.cn/CottonMD/download.1	3-79_HAU	*Gossypium barbadense*	Gbar_D11G036920.2	GbUBC117
https://yanglab.hzau.edu.cn/CottonMD/download.1	3-79_HAU	*Gossypium barbadense*	Gbar_D12G003560.1	GbUBC118
https://yanglab.hzau.edu.cn/CottonMD/download.1	3-79_HAU	*Gossypium barbadense*	Gbar_D12G006400.1	GbUBC119
https://yanglab.hzau.edu.cn/CottonMD/download.1	3-79_HAU	*Gossypium barbadense*	Gbar_D12G006820.1	GbUBC120
https://yanglab.hzau.edu.cn/CottonMD/download.1	3-79_HAU	*Gossypium barbadense*	Gbar_D12G010490.1	GbUBC121
https://yanglab.hzau.edu.cn/CottonMD/download.1	3-79_HAU	*Gossypium barbadense*	Gbar_D12G020750.1	GbUBC122
https://yanglab.hzau.edu.cn/CottonMD/download.1	3-79_HAU	*Gossypium barbadense*	Gbar_D12G024010.1	GbUBC123
https://yanglab.hzau.edu.cn/CottonMD/download.1	3-79_HAU	*Gossypium barbadense*	Gbar_D12G026270.1	GbUBC124
https://yanglab.hzau.edu.cn/CottonMD/download.1	3-79_HAU	*Gossypium barbadense*	Gbar_D13G024860.1	GbUBC125
https://yanglab.hzau.edu.cn/CottonMD/download.1	TM1_NBI	*Gossypium hirsutum*	Gh_A01G0287	Gh_A01G0287
https://yanglab.hzau.edu.cn/CottonMD/download.1	TM1_NBI	*Gossypium hirsutum*	Gh_A01G0391	Gh_A01G0391

https://yanglab.hzau.edu.cn/CottonMD/download.1	TM1_NBI	*Gossypium hirsutum*	Gh_A11G2595	Gh_A11G2595
https://yanglab.hzau.edu.cn/CottonMD/download.1	TM1_NBI	*Gossypium hirsutum*	Gh_A13G1276	Gh_A13G1276
https://yanglab.hzau.edu.cn/CottonMD/download.1	TM1_NBI	*Gossypium hirsutum*	Gh_D01G0328	Gh_D01G0328
https://yanglab.hzau.edu.cn/CottonMD/download.1	TM1_NBI	*Gossypium hirsutum*	Gh_D01G2341	Gh_D01G2341
https://yanglab.hzau.edu.cn/CottonMD/download.1	TM1_NBI	*Gossypium hirsutum*	Gh_D11G2962	Gh_D11G2962
https://yanglab.hzau.edu.cn/CottonMD/download.1	TM1_NBI	*Gossypium hirsutum*	Gh_D13G1580	Gh_D13G1580
https://yanglab.hzau.edu.cn/CottonMD/download.1	TM1_NBI	*Gossypium hirsutum*	Gh_A04G0340	Gh_A04G0340
https://yanglab.hzau.edu.cn/CottonMD/download.1	TM1_NBI	*Gossypium hirsutum*	Gh_D05G3306	Gh_D05G3306
https://yanglab.hzau.edu.cn/CottonMD/download.1	TM1_NBI	*Gossypium hirsutum*	Gh_D06G0990	Gh_D06G0990
https://yanglab.hzau.edu.cn/CottonMD/download.1	TM1_NBI	*Gossypium hirsutum*	Gh_A06G0852	Gh_A06G0852
https://yanglab.hzau.edu.cn/CottonMD/download.1	TM1_NBI	*Gossypium hirsutum*	Gh_A07G0171	Gh_A07G0171
https://yanglab.hzau.edu.cn/CottonMD/download.1	TM1_NBI	*Gossypium hirsutum*	Gh_D07G0228	Gh_D07G0228
https://yanglab.hzau.edu.cn/CottonMD/download.1	TM1_NBI	*Gossypium hirsutum*	Gh_A04G1233	Gh_A04G1233
https://yanglab.hzau.edu.cn/CottonMD/download.1	TM1_NBI	*Gossypium hirsutum*	Gh_D04G1856	Gh_D04G1856
https://yanglab.hzau.edu.cn/CottonMD/download.1	TM1_NBI	*Gossypium hirsutum*	Gh_A03G1030	Gh_A03G1030
https://yanglab.hzau.edu.cn/CottonMD/download.1	TM1_NBI	*Gossypium hirsutum*	Gh_D02G1422	Gh_D02G1422
https://yanglab.hzau.edu.cn/CottonMD/download.1	TM1_NBI	*Gossypium hirsutum*	Gh_D01G1399	Gh_D01G1399
https://yanglab.hzau.edu.cn/CottonMD/download.1	TM1_NBI	*Gossypium hirsutum*	Gh_A01G1222	Gh_A01G1222
https://yanglab.hzau.edu.cn/CottonMD/download.1	TM1_NBI	*Gossypium hirsutum*	Gh_D11G3214	Gh_D11G3214
https://yanglab.hzau.edu.cn/CottonMD/download.1	TM1_NBI	*Gossypium hirsutum*	Gh_D01G1981	Gh_D01G1981
https://yanglab.hzau.edu.cn/CottonMD/download.1	TM1_NBI	*Gossypium hirsutum*	Gh_D11G3212	Gh_D11G3212
https://yanglab.hzau.edu.cn/CottonMD/download.1	TM1_NBI	*Gossypium hirsutum*	Gh_D11G3213	Gh_D11G3213
https://yanglab.hzau.edu.cn/CottonMD/download.1	TM1_NBI	*Gossypium hirsutum*	Gh_A01G1733	Gh_A01G1733
https://yanglab.hzau.edu.cn/CottonMD/download.1	TM1_NBI	*Gossypium hirsutum*	Gh_A11G2730	Gh_A11G2730
https://yanglab.hzau.edu.cn/CottonMD/download.1	TM1_NBI	*Gossypium hirsutum*	Gh_sca005098G02	Gh_sca005098G02
https://yanglab.hzau.edu.cn/CottonMD/download.1	TM1_NBI	*Gossypium hirsutum*	Gh_D05G0946	Gh_D05G0946
https://yanglab.hzau.edu.cn/CottonMD/download.1	TM1_NBI	*Gossypium hirsutum*	Gh_A05G0825	Gh_A05G0825
https://yanglab.hzau.edu.cn/CottonMD/download.1	TM1_NBI	*Gossypium hirsutum*	Gh_A13G1984	Gh_A13G1984
https://yanglab.hzau.edu.cn/CottonMD/download.1	TM1_NBI	*Gossypium hirsutum*	Gh_D13G2383	Gh_D13G2383
https://yanglab.hzau.edu.cn/CottonMD/download.1	TM1_NBI	*Gossypium hirsutum*	Gh_A03G0346	Gh_A03G0346
https://yanglab.hzau.edu.cn/CottonMD/download.1	TM1_NBI	*Gossypium hirsutum*	Gh_A07G0881	Gh_A07G0881
https://yanglab.hzau.edu.cn/CottonMD/download.1	TM1_NBI	*Gossypium hirsutum*	Gh_D03G1230	Gh_D03G1230
https://yanglab.hzau.edu.cn/CottonMD/download.1	TM1_NBI	*Gossypium hirsutum*	Gh_D07G0950	Gh_D07G0950
https://yanglab.hzau.edu.cn/CottonMD/download.1	TM1_NBI	*Gossypium hirsutum*	Gh_D08G1655	Gh_D08G1655
https://yanglab.hzau.edu.cn/CottonMD/download.1	TM1_NBI	*Gossypium hirsutum*	Gh_A08G1359	Gh_A08G1359
https://yanglab.hzau.edu.cn/CottonMD/download.1	TM1_NBI	*Gossypium hirsutum*	Gh_A05G1771	Gh_A05G1771
https://yanglab.hzau.edu.cn/CottonMD/download.1	TM1_NBI	*Gossypium hirsutum*	Gh_D05G1965	Gh_D05G1965
https://yanglab.hzau.edu.cn/CottonMD/download.1	TM1_NBI	*Gossypium hirsutum*	Gh_A11G1797	Gh_A11G1797
https://yanglab.hzau.edu.cn/CottonMD/download.1	TM1_NBI	*Gossypium hirsutum*	Gh_D11G1959	Gh_D11G1959
https://yanglab.hzau.edu.cn/CottonMD/download.1	TM1_NBI	*Gossypium hirsutum*	Gh_D08G2323	Gh_D08G2323
https://yanglab.hzau.edu.cn/CottonMD/download.1	TM1_NBI	*Gossypium hirsutum*	Gh_A08G2339	Gh_A08G2339
https://yanglab.hzau.edu.cn/CottonMD/download.1	TM1_NBI	*Gossypium hirsutum*	Gh_A12G1595	Gh_A12G1595
https://yanglab.hzau.edu.cn/CottonMD/download.1	TM1_NBI	*Gossypium hirsutum*	Gh_D12G1735	Gh_D12G1735

https://yanglab.hzau.edu.cn/CottonMD/download.1	TM1_NBI	*Gossypium hirsutum*	Gh_A11G2066	Gh_A11G2066
https://yanglab.hzau.edu.cn/CottonMD/download.1	TM1_NBI	*Gossypium hirsutum*	Gh_A12G1527	Gh_A12G1527
https://yanglab.hzau.edu.cn/CottonMD/download.1	TM1_NBI	*Gossypium hirsutum*	Gh_D11G2370	Gh_D11G2370
https://yanglab.hzau.edu.cn/CottonMD/download.1	TM1_NBI	*Gossypium hirsutum*	Gh_D12G1650	Gh_D12G1650
https://yanglab.hzau.edu.cn/CottonMD/download.1	TM1_NBI	*Gossypium hirsutum*	Gh_D04G0024	Gh_D04G0024
https://yanglab.hzau.edu.cn/CottonMD/download.1	TM1_NBI	*Gossypium hirsutum*	Gh_A05G3581	Gh_A05G3581
https://yanglab.hzau.edu.cn/CottonMD/download.1	TM1_NBI	*Gossypium hirsutum*	Gh_A05G2558	Gh_A05G2558
https://yanglab.hzau.edu.cn/CottonMD/download.1	TM1_NBI	*Gossypium hirsutum*	Gh_D07G1216	Gh_D07G1216
https://yanglab.hzau.edu.cn/CottonMD/download.1	TM1_NBI	*Gossypium hirsutum*	Gh_A07G2249	Gh_A07G2249
https://yanglab.hzau.edu.cn/CottonMD/download.1	TM1_NBI	*Gossypium hirsutum*	Gh_D06G1165	Gh_D06G1165
https://yanglab.hzau.edu.cn/CottonMD/download.1	TM1_NBI	*Gossypium hirsutum*	Gh_A06G0934	Gh_A06G0934
https://yanglab.hzau.edu.cn/CottonMD/download.1	TM1_NBI	*Gossypium hirsutum*	Gh_A10G2225	Gh_A10G2225
https://yanglab.hzau.edu.cn/CottonMD/download.1	TM1_NBI	*Gossypium hirsutum*	Gh_D06G1563	Gh_D06G1563
https://yanglab.hzau.edu.cn/CottonMD/download.1	TM1_NBI	*Gossypium hirsutum*	Gh_D10G0484	Gh_D10G0484
https://yanglab.hzau.edu.cn/CottonMD/download.1	TM1_NBI	*Gossypium hirsutum*	Gh_A06G1244	Gh_A06G1244
https://yanglab.hzau.edu.cn/CottonMD/download.1	TM1_NBI	*Gossypium hirsutum*	Gh_A10G1526	Gh_A10G1526
https://yanglab.hzau.edu.cn/CottonMD/download.1	TM1_NBI	*Gossypium hirsutum*	Gh_D09G2267	Gh_D09G2267
https://yanglab.hzau.edu.cn/CottonMD/download.1	TM1_NBI	*Gossypium hirsutum*	Gh_D10G1777	Gh_D10G1777
https://yanglab.hzau.edu.cn/CottonMD/download.1	TM1_NBI	*Gossypium hirsutum*	Gh_A05G2353	Gh_A05G2353
https://yanglab.hzau.edu.cn/CottonMD/download.1	TM1_NBI	*Gossypium hirsutum*	Gh_D05G2620	Gh_D05G2620
https://yanglab.hzau.edu.cn/CottonMD/download.1	TM1_NBI	*Gossypium hirsutum*	Gh_A11G1594	Gh_A11G1594
https://yanglab.hzau.edu.cn/CottonMD/download.1	TM1_NBI	*Gossypium hirsutum*	Gh_D11G1752	Gh_D11G1752
https://yanglab.hzau.edu.cn/CottonMD/download.1	TM1_NBI	*Gossypium hirsutum*	Gh_D10G2102	Gh_D10G2102
https://yanglab.hzau.edu.cn/CottonMD/download.1	TM1_NBI	*Gossypium hirsutum*	Gh_D10G2103	Gh_D10G2103
https://yanglab.hzau.edu.cn/CottonMD/download.1	TM1_NBI	*Gossypium hirsutum*	Gh_D10G1113	Gh_D10G1113
https://yanglab.hzau.edu.cn/CottonMD/download.1	TM1_NBI	*Gossypium hirsutum*	Gh_D11G0229	Gh_D11G0229
https://yanglab.hzau.edu.cn/CottonMD/download.1	TM1_NBI	*Gossypium hirsutum*	Gh_A10G1357	Gh_A10G1357
https://yanglab.hzau.edu.cn/CottonMD/download.1	TM1_NBI	*Gossypium hirsutum*	Gh_D08G0303	Gh_D08G0303
https://yanglab.hzau.edu.cn/CottonMD/download.1	TM1_NBI	*Gossypium hirsutum*	Gh_A08G0223	Gh_A08G0223
https://yanglab.hzau.edu.cn/CottonMD/download.1	TM1_NBI	*Gossypium hirsutum*	Gh_A04G0010	Gh_A04G0010
https://yanglab.hzau.edu.cn/CottonMD/download.1	TM1_NBI	*Gossypium hirsutum*	Gh_D09G2059	Gh_D09G2059
https://yanglab.hzau.edu.cn/CottonMD/download.1	TM1_NBI	*Gossypium hirsutum*	Gh_A09G2270	Gh_A09G2270
https://yanglab.hzau.edu.cn/CottonMD/download.1	TM1_NBI	*Gossypium hirsutum*	Gh_D02G0037	Gh_D02G0037
https://yanglab.hzau.edu.cn/CottonMD/download.1	TM1_NBI	*Gossypium hirsutum*	Gh_A12G2706	Gh_A12G2706
https://yanglab.hzau.edu.cn/CottonMD/download.1	TM1_NBI	*Gossypium hirsutum*	Gh_D12G2684	Gh_D12G2684
https://yanglab.hzau.edu.cn/CottonMD/download.1	TM1_NBI	*Gossypium hirsutum*	Gh_A02G0022	Gh_A02G0022
https://yanglab.hzau.edu.cn/CottonMD/download.1	TM1_NBI	*Gossypium hirsutum*	Gh_A08G2435	Gh_A08G2435
https://yanglab.hzau.edu.cn/CottonMD/download.1	TM1_NBI	*Gossypium hirsutum*	Gh_A11G0215	Gh_A11G0215
https://yanglab.hzau.edu.cn/CottonMD/download.1	TM1_NBI	*Gossypium hirsutum*	Gh_D08G1242	Gh_D08G1242
https://yanglab.hzau.edu.cn/CottonMD/download.1	TM1_NBI	*Gossypium hirsutum*	Gh_A11G0853	Gh_A11G0853
https://yanglab.hzau.edu.cn/CottonMD/download.1	TM1_NBI	*Gossypium hirsutum*	Gh_A02G1007	Gh_A02G1007
https://yanglab.hzau.edu.cn/CottonMD/download.1	TM1_NBI	*Gossypium hirsutum*	Gh_A11G0117	Gh_A11G0117
https://yanglab.hzau.edu.cn/CottonMD/download.1	TM1_NBI	*Gossypium hirsutum*	Gh_D03G0718	Gh_D03G0718

https://yanglab.hzau.edu.cn/CottonMD/download.1	TM1_NBI	*Gossypium hirsutum*	Gh_D11G0131	Gh_D11G0131
https://yanglab.hzau.edu.cn/CottonMD/download.1	TM1_NBI	*Gossypium hirsutum*	Gh_D09G1736	Gh_D09G1736
https://yanglab.hzau.edu.cn/CottonMD/download.1	TM1_NBI	*Gossypium hirsutum*	Gh_D02G0036	Gh_D02G0036
https://yanglab.hzau.edu.cn/CottonMD/download.1	TM1_NBI	*Gossypium hirsutum*	Gh_A09G1642	Gh_A09G1642
https://yanglab.hzau.edu.cn/CottonMD/download.1	TM1_NBI	*Gossypium hirsutum*	Gh_A02G0023	Gh_A02G0023
https://yanglab.hzau.edu.cn/CottonMD/download.1	TM1_NBI	*Gossypium hirsutum*	Gh_D11G0995	Gh_D11G0995
https://yanglab.hzau.edu.cn/CottonMD/download.1	TM1_NBI	*Gossypium hirsutum*	Gh_D12G0320	Gh_D12G0320
https://yanglab.hzau.edu.cn/CottonMD/download.1	TM1_NBI	*Gossypium hirsutum*	Gh_A12G0342	Gh_A12G0342
https://yanglab.hzau.edu.cn/CottonMD/download.1	TM1_NBI	*Gossypium hirsutum*	Gh_D01G1637	Gh_D01G1637
https://yanglab.hzau.edu.cn/CottonMD/download.1	TM1_NBI	*Gossypium hirsutum*	Gh_A01G1393	Gh_A01G1393
https://yanglab.hzau.edu.cn/CottonMD/download.1	TM1_NBI	*Gossypium hirsutum*	Gh_A12G1751	Gh_A12G1751
https://yanglab.hzau.edu.cn/CottonMD/download.1	TM1_NBI	*Gossypium hirsutum*	Gh_D12G1902	Gh_D12G1902
https://yanglab.hzau.edu.cn/CottonMD/download.1	TM1_NBI	*Gossypium hirsutum*	Gh_D11G0407	Gh_D11G0407
https://yanglab.hzau.edu.cn/CottonMD/download.1	TM1_NBI	*Gossypium hirsutum*	Gh_A11G0350	Gh_A11G0350
https://yanglab.hzau.edu.cn/CottonMD/download.1	TM1_NBI	*Gossypium hirsutum*	Gh_D06G0591	Gh_D06G0591
https://yanglab.hzau.edu.cn/CottonMD/download.1	TM1_NBI	*Gossypium hirsutum*	Gh_A06G0535	Gh_A06G0535
https://yanglab.hzau.edu.cn/CottonMD/download.1	TM1_NBI	*Gossypium hirsutum*	Gh_D12G0587	Gh_D12G0587
https://yanglab.hzau.edu.cn/CottonMD/download.1	TM1_NBI	*Gossypium hirsutum*	Gh_A12G0572	Gh_A12G0572
https://yanglab.hzau.edu.cn/CottonMD/download.1	TM1_NBI	*Gossypium hirsutum*	Gh_D03G0112	Gh_D03G0112
https://yanglab.hzau.edu.cn/CottonMD/download.1	TM1_NBI	*Gossypium hirsutum*	Gh_A02G1610	Gh_A02G1610
https://yanglab.hzau.edu.cn/CottonMD/download.1	TM1_NBI	*Gossypium hirsutum*	Gh_A01G0928	Gh_A01G0928
https://yanglab.hzau.edu.cn/CottonMD/download.1	TM1_NBI	*Gossypium hirsutum*	Gh_D05G1814	Gh_D05G1814
https://yanglab.hzau.edu.cn/CottonMD/download.1	TM1_NBI	*Gossypium hirsutum*	Gh_D01G0973	Gh_D01G0973
https://yanglab.hzau.edu.cn/CottonMD/download.1	TM1_NBI	*Gossypium hirsutum*	Gh_A05G1632	Gh_A05G1632
https://yanglab.hzau.edu.cn/CottonMD/download.1	TM1_NBI	*Gossypium hirsutum*	Gh_D06G1901	Gh_D06G1901
https://yanglab.hzau.edu.cn/CottonMD/download.1	TM1_NBI	*Gossypium hirsutum*	Gh_A06G1532	Gh_A06G1532
https://yanglab.hzau.edu.cn/CottonMD/download.1	TM1_NBI	*Gossypium hirsutum*	Gh_D10G1977	Gh_D10G1977
https://yanglab.hzau.edu.cn/CottonMD/download.1	TM1_NBI	*Gossypium hirsutum*	Gh_A10G1708	Gh_A10G1708
https://yanglab.hzau.edu.cn/CottonMD/download.1	TM1_NBI	*Gossypium hirsutum*	Gh_D07G2119	Gh_D07G2119
https://yanglab.hzau.edu.cn/CottonMD/download.1	TM1_NBI	*Gossypium hirsutum*	Gh_A07G1902	Gh_A07G1902
https://yanglab.hzau.edu.cn/CottonMD/download.1	TM1_NBI	*Gossypium hirsutum*	Gh_D09G1617	Gh_D09G1617
https://yanglab.hzau.edu.cn/CottonMD/download.1	TM1_NBI	*Gossypium hirsutum*	Gh_A09G1546	Gh_A09G1546
https://yanglab.hzau.edu.cn/CottonMD/download.1	TM1_NBI	*Gossypium hirsutum*	Gh_D07G0619	Gh_D07G0619
https://yanglab.hzau.edu.cn/CottonMD/download.1	TM1_NBI	*Gossypium hirsutum*	Gh_A07G0553	Gh_A07G0553
https://yanglab.hzau.edu.cn/CottonMD/download.1	TM1_NBI	*Gossypium hirsutum*	Gh_D12G2275	Gh_D12G2275
https://yanglab.hzau.edu.cn/CottonMD/download.1	TM1_NBI	*Gossypium hirsutum*	Gh_A12G2103	Gh_A12G2103
https://yanglab.hzau.edu.cn/CottonMD/download.1	TM1_NBI	*Gossypium hirsutum*	Gh_D03G1436	Gh_D03G1436
https://yanglab.hzau.edu.cn/CottonMD/download.1	TM1_NBI	*Gossypium hirsutum*	Gh_D11G0215	Gh_D11G0215
https://yanglab.hzau.edu.cn/CottonMD/download.1	TM1_NBI	*Gossypium hirsutum*	Gh_A11G0204	Gh_A11G0204
https://yanglab.hzau.edu.cn/CottonMD/download.1	TM1_NBI	*Gossypium hirsutum*	Gh_A03G0143	Gh_A03G0143
https://yanglab.hzau.edu.cn/CottonMD/download.1	TM1_NBI	*Gossypium hirsutum*	Gh_D09G2260	Gh_D09G2260
https://yanglab.hzau.edu.cn/CottonMD/download.1	TM1_NBI	*Gossypium hirsutum*	Gh_A09G2053	Gh_A09G2053
https://yanglab.hzau.edu.cn/CottonMD/download.1	TM1_NBI	*Gossypium hirsutum*	Gh_D10G0234	Gh_D10G0234

https://yanglab.hzau.edu.cn/CottonMD/download.1	TM1_NBI	*Gossypium hirsutum*	Gh_A10G0253	Gh_A10G0253
https://yanglab.hzau.edu.cn/CottonMD/download.1	TM1_NBI	*Gossypium hirsutum*	Gh_D06G1375	Gh_D06G1375
https://yanglab.hzau.edu.cn/CottonMD/download.1	TM1_NBI	*Gossypium hirsutum*	Gh_A06G1121	Gh_A06G1121
https://yanglab.hzau.edu.cn/CottonMD/download.1	TM1_NBI	*Gossypium hirsutum*	Gh_A04G1472	Gh_A04G1472
https://yanglab.hzau.edu.cn/CottonMD/download.1	TM1_NBI	*Gossypium hirsutum*	Gh_D04G1652	Gh_D04G1652
https://yanglab.hzau.edu.cn/CottonMD/download.1	TM1_NBI	*Gossypium hirsutum*	Gh_D02G1828	Gh_D02G1828
https://yanglab.hzau.edu.cn/CottonMD/download.1	TM1_NBI	*Gossypium hirsutum*	Gh_A03G1388	Gh_A03G1388
https://yanglab.hzau.edu.cn/CottonMD/download.1	TM1_NBI	*Gossypium hirsutum*	Gh_D01G0169	Gh_D01G0169
https://yanglab.hzau.edu.cn/CottonMD/download.1	TM1_NBI	*Gossypium hirsutum*	Gh_A01G0121	Gh_A01G0121
https://yanglab.hzau.edu.cn/CottonMD/download.1	TM1_NBI	*Gossypium hirsutum*	Gh_D11G2938	Gh_D11G2938
https://yanglab.hzau.edu.cn/CottonMD/download.1	TM1_NBI	*Gossypium hirsutum*	Gh_A11G2565	Gh_A11G2565
https://yanglab.hzau.edu.cn/CottonMD/download.1	TM1_NBI	*Gossypium hirsutum*	Gh_D01G0475	Gh_D01G0475
https://yanglab.hzau.edu.cn/CottonMD/download.1	TM1_NBI	*Gossypium hirsutum*	Gh_A01G0466	Gh_A01G0466
https://yanglab.hzau.edu.cn/CottonMD/download.1	TM1_NBI	*Gossypium hirsutum*	Gh_Sca079762G01	Gh_Sca079762G01
https://yanglab.hzau.edu.cn/CottonMD/download.1	TM1_NBI	*Gossypium hirsutum*	Gh_D12G0626	Gh_D12G0626
https://yanglab.hzau.edu.cn/CottonMD/download.1	TM1_NBI	*Gossypium hirsutum*	Gh_A12G0614	Gh_A12G0614
https://yanglab.hzau.edu.cn/CottonMD/download.1	TM1_NBI	*Gossypium hirsutum*	Gh_D11G1882	Gh_D11G1882
https://yanglab.hzau.edu.cn/CottonMD/download.1	TM1_NBI	*Gossypium hirsutum*	Gh_A11G1724	Gh_A11G1724
https://yanglab.hzau.edu.cn/CottonMD/download.1	TM1_NBI	*Gossypium hirsutum*	Gh_D11G0474	Gh_D11G0474
https://yanglab.hzau.edu.cn/CottonMD/download.1	TM1_NBI	*Gossypium hirsutum*	Gh_A11G0412	Gh_A11G0412
https://yanglab.hzau.edu.cn/CottonMD/download.1	TM1_NBI	*Gossypium hirsutum*	Gh_A05G2262	Gh_A05G2262
https://yanglab.hzau.edu.cn/CottonMD/download.1	TM1_NBI	*Gossypium hirsutum*	Gh_D05G2520	Gh_D05G2520
https://yanglab.hzau.edu.cn/CottonMD/download.1	TM1_NBI	*Gossypium hirsutum*	Gh_D06G2321	Gh_D06G2321
https://yanglab.hzau.edu.cn/CottonMD/download.1	TM1_NBI	*Gossypium hirsutum*	Gh_A06G0237	Gh_A06G0237
https://yanglab.hzau.edu.cn/CottonMD/download.1	TM1_NBI	*Gossypium hirsutum*	Gh_D11G0026	Gh_D11G0026
https://yanglab.hzau.edu.cn/CottonMD/download.1	TM1_NBI	*Gossypium hirsutum*	Gh_A11G0028	Gh_A11G0028
https://yanglab.hzau.edu.cn/CottonMD/download.1	TM1_NBI	*Gossypium hirsutum*	Gh_A01G2127	Gh_A01G2127
https://yanglab.hzau.edu.cn/CottonMD/download.1	TM1_NBI	*Gossypium hirsutum*	Gh_D01G1394	Gh_D01G1394
https://yanglab.hzau.edu.cn/CottonMD/download.1	TM1_NBI	*Gossypium hirsutum*	Gh_A08G1860	Gh_A08G1860
https://yanglab.hzau.edu.cn/CottonMD/download.1	TM1_NBI	*Gossypium hirsutum*	Gh_D08G2221	Gh_D08G2221
https://yanglab.hzau.edu.cn/CottonMD/download.1	TM1_NBI	*Gossypium hirsutum*	Gh_D11G2726	Gh_D11G2726
https://yanglab.hzau.edu.cn/CottonMD/download.1	TM1_NBI	*Gossypium hirsutum*	Gh_A11G2410	Gh_A11G2410
https://yanglab.hzau.edu.cn/CottonMD/download.1	TM1_NBI	*Gossypium hirsutum*	Gh_A02G1128	Gh_A02G1128
https://yanglab.hzau.edu.cn/CottonMD/download.1	TM1_NBI	*Gossypium hirsutum*	Gh_D03G0551	Gh_D03G0551
https://yanglab.hzau.edu.cn/CottonMD/download.1	TM1_NBI	*Gossypium hirsutum*	Gh_D12G0965	Gh_D12G0965
https://yanglab.hzau.edu.cn/CottonMD/download.1	TM1_NBI	*Gossypium hirsutum*	Gh_A12G0882	Gh_A12G0882
https://yanglab.hzau.edu.cn/CottonMD/download.1	TM1_NBI	*Gossypium hirsutum*	Gh_A11G1674	Gh_A11G1674
https://yanglab.hzau.edu.cn/CottonMD/download.1	TM1_NBI	*Gossypium hirsutum*	Gh_D11G1831	Gh_D11G1831
https://www.arabidopsis.org/download/list?dir=Proteins%2FAraport11_protein_lists	Araport11_pep_20220914	*Arabidopsis*	AT1G27530.1	AT1G27530.1
https://www.arabidopsis.org/download/list?dir=Proteins%2FAraport11_protein_lists	Araport11_pep_20220914	*Arabidopsis*	AT1G51730.1	AT1G51730.1
https://www.arabidopsis.org/download/list?dir=Proteins%2FAraport11_protein_lists	Araport11_pep_20220914	*Arabidopsis*	AT1G53023.1	AT1G53023.1
https://www.arabidopsis.org/download/list?dir=Proteins%2FAraport11_protein_lists	Araport11_pep_20220914	*Arabidopsis*	AT2G18600.1	AT2G18600.1
https://www.arabidopsis.org/download/list?dir=Proteins%2FAraport11_protein_lists	Araport11_pep_20220914	*Arabidopsis*	AT2G38830.1	AT2G38830.1

https://www.arabidopsis.org/download/list?dir=Proteins%2FAraport11_protein_lists	Araport11_pep_20220914	*Arabidopsis*	AT3G26750.1	AT3G26750.1
https://www.arabidopsis.org/download/list?dir=Proteins%2FAraport11_protein_lists	Araport11_pep_20220914	*Arabidopsis*	AT2G32790.1	AT2G32790.1
https://www.arabidopsis.org/download/list?dir=Proteins%2FAraport11_protein_lists	Araport11_pep_20220914	*Arabidopsis*	AT5G13860.1	ELC-Like
https://www.arabidopsis.org/download/list?dir=Proteins%2FAraport11_protein_lists	Araport11_pep_20220914	*Arabidopsis*	AT3G13550.1	AtCOP10
https://www.arabidopsis.org/download/list?dir=Proteins%2FAraport11_protein_lists	Araport11_pep_20220914	*Arabidopsis*	AT3G12400.1	AtELC
https://www.arabidopsis.org/download/list?dir=Proteins%2FAraport11_protein_lists	Araport11_pep_20220914	*Arabidopsis*	AT4G36800.1	AtRCE1
https://www.arabidopsis.org/download/list?dir=Proteins%2FAraport11_protein_lists	Araport11_pep_20220914	*Arabidopsis*	AT3G57870.1	AtSCE1
https://www.arabidopsis.org/download/list?dir=Proteins%2FAraport11_protein_lists	Araport11_pep_20220914	*Arabidopsis*	AT1G14400.1	AtUBC1
https://www.arabidopsis.org/download/list?dir=Proteins%2FAraport11_protein_lists	Araport11_pep_20220914	*Arabidopsis*	AT2G02760.1	AtUBC2
https://www.arabidopsis.org/download/list?dir=Proteins%2FAraport11_protein_lists	Araport11_pep_20220914	*Arabidopsis*	AT5G62540.1	AtUBC3
https://www.arabidopsis.org/download/list?dir=Proteins%2FAraport11_protein_lists	Araport11_pep_20220914	*Arabidopsis*	AT5G41340.1	AtUBC4
https://www.arabidopsis.org/download/list?dir=Proteins%2FAraport11_protein_lists	Araport11_pep_20220914	*Arabidopsis*	AT1G63800.1	AtUBC5
https://www.arabidopsis.org/download/list?dir=Proteins%2FAraport11_protein_lists	Araport11_pep_20220914	*Arabidopsis*	AT2G46030.1	AtUBC6
https://www.arabidopsis.org/download/list?dir=Proteins%2FAraport11_protein_lists	Araport11_pep_20220914	*Arabidopsis*	AT5G50870.1	AtUBC7
https://www.arabidopsis.org/download/list?dir=Proteins%2FAraport11_protein_lists	Araport11_pep_20220914	*Arabidopsis*	AT5G41700.1	AtUBC8
https://www.arabidopsis.org/download/list?dir=Proteins%2FAraport11_protein_lists	Araport11_pep_20220914	*Arabidopsis*	AT4G27960.1	AtUBC9
https://www.arabidopsis.org/download/list?dir=Proteins%2FAraport11_protein_lists	Araport11_pep_20220914	*Arabidopsis*	AT5G53300.1	AtUBC10
https://www.arabidopsis.org/download/list?dir=Proteins%2FAraport11_protein_lists	Araport11_pep_20220914	*Arabidopsis*	AT3G08690.1	AtUBC11
https://www.arabidopsis.org/download/list?dir=Proteins%2FAraport11_protein_lists	Araport11_pep_20220914	*Arabidopsis*	AT3G08700.1	AtUBC12
https://www.arabidopsis.org/download/list?dir=Proteins%2FAraport11_protein_lists	Araport11_pep_20220914	*Arabidopsis*	AT3G46460.1	AtUBC13
https://www.arabidopsis.org/download/list?dir=Proteins%2FAraport11_protein_lists	Araport11_pep_20220914	*Arabidopsis*	AT3G55380.1	AtUBC14
https://www.arabidopsis.org/download/list?dir=Proteins%2FAraport11_protein_lists	Araport11_pep_20220914	*Arabidopsis*	AT1G45050.1	AtUBC15
https://www.arabidopsis.org/download/list?dir=Proteins%2FAraport11_protein_lists	Araport11_pep_20220914	*Arabidopsis*	AT1G75440.1	AtUBC16
https://www.arabidopsis.org/download/list?dir=Proteins%2FAraport11_protein_lists	Araport11_pep_20220914	*Arabidopsis*	AT4G36410.1	AtUBC17
https://www.arabidopsis.org/download/list?dir=Proteins%2FAraport11_protein_lists	Araport11_pep_20220914	*Arabidopsis*	AT5G42990.1	AtUBC18
https://www.arabidopsis.org/download/list?dir=Proteins%2FAraport11_protein_lists	Araport11_pep_20220914	*Arabidopsis*	AT3G20060.1	AtUBC19
https://www.arabidopsis.org/download/list?dir=Proteins%2FAraport11_protein_lists	Araport11_pep_20220914	*Arabidopsis*	AT1G50490.1	AtUBC20
https://www.arabidopsis.org/download/list?dir=Proteins%2FAraport11_protein_lists	Araport11_pep_20220914	*Arabidopsis*	AT5G25760.1	AtUBC21
https://www.arabidopsis.org/download/list?dir=Proteins%2FAraport11_protein_lists	Araport11_pep_20220914	*Arabidopsis*	AT5G05080.1	AtUBC22
https://www.arabidopsis.org/download/list?dir=Proteins%2FAraport11_protein_lists	Araport11_pep_20220914	*Arabidopsis*	AT2G16920.1	AtUBC23
https://www.arabidopsis.org/download/list?dir=Proteins%2FAraport11_protein_lists	Araport11_pep_20220914	*Arabidopsis*	AT2G33770.1	AtUBC24
https://www.arabidopsis.org/download/list?dir=Proteins%2FAraport11_protein_lists	Araport11_pep_20220914	*Arabidopsis*	AT3G15355.1	AtUBC25
https://www.arabidopsis.org/download/list?dir=Proteins%2FAraport11_protein_lists	Araport11_pep_20220914	*Arabidopsis*	AT1G53025.1	AtUBC26
https://www.arabidopsis.org/download/list?dir=Proteins%2FAraport11_protein_lists	Araport11_pep_20220914	*Arabidopsis*	AT5G59300.1	AtUBC27
https://www.arabidopsis.org/download/list?dir=Proteins%2FAraport11_protein_lists	Araport11_pep_20220914	*Arabidopsis*	AT1G64230.1	AtUBC28
https://www.arabidopsis.org/download/list?dir=Proteins%2FAraport11_protein_lists	Araport11_pep_20220914	*Arabidopsis*	AT2G16740.1	AtUBC29
https://www.arabidopsis.org/download/list?dir=Proteins%2FAraport11_protein_lists	Araport11_pep_20220914	*Arabidopsis*	AT5G56150.1	AtUBC30
https://www.arabidopsis.org/download/list?dir=Proteins%2FAraport11_protein_lists	Araport11_pep_20220914	*Arabidopsis*	AT1G36340.1	AtUBC31
https://www.arabidopsis.org/download/list?dir=Proteins%2FAraport11_protein_lists	Araport11_pep_20220914	*Arabidopsis*	AT3G17000.1	AtUBC32
https://www.arabidopsis.org/download/list?dir=Proteins%2FAraport11_protein_lists	Araport11_pep_20220914	*Arabidopsis*	AT5G50430.1	AtUBC33
https://www.arabidopsis.org/download/list?dir=Proteins%2FAraport11_protein_lists	Araport11_pep_20220914	*Arabidopsis*	AT1G17280.1	AtUBC34
https://www.arabidopsis.org/download/list?dir=Proteins%2FAraport11_protein_lists	Araport11_pep_20220914	*Arabidopsis*	AT1G78870.1	AtUBC35
https://www.arabidopsis.org/download/list?dir=Proteins%2FAraport11_protein_lists	Araport11_pep_20220914	*Arabidopsis*	AT1G16890.1	AtUBC36

https://www.arabidopsis.org/download/list?dir=Proteins%2FAraport11_protein_lists	Araport11_pep_20220914	*Arabidopsis*	AT3G24515.1	AtUBC37
https://www.arabidopsis.org/download/list?dir=Proteins%2FAraport11_protein_lists	Araport11_pep_20220914	*Arabidopsis*	AT1G23260.1	AtUEV1A
https://www.arabidopsis.org/download/list?dir=Proteins%2FAraport11_protein_lists	Araport11_pep_20220914	*Arabidopsis*	AT1G70660.1	AtUEV1B
https://www.arabidopsis.org/download/list?dir=Proteins%2FAraport11_protein_lists	Araport11_pep_20220914	*Arabidopsis*	AT2G36060.1	AtUEV1C
https://www.arabidopsis.org/download/list?dir=Proteins%2FAraport11_protein_lists	Araport11_pep_20220914	*Arabidopsis*	AT3G52560.1	AtUEV1D
