# Supplementary material for: Genome-Wide Identification of the GbUBC Gene Family in Sea-Island Cotton (Gossypium barbadense) and the Active Regulation of Drought Resistance in Cotton by GbUBC23
Source: Int J Mol Sci. 2024 Dec 2;25(23):12948. doi: 10.3390/ijms252312948 (PMC11640981; doi:10.3390/ijms252312948)
Supplement: Supplementary file 1 [file ijms-25-12948-s001.zip › Table S2.pdf]

Table S2 Physicochemical properties of the GbUBC family members

Gene	Gen ID	CDS Length (bp)	Amino Acids (aa)	Molecular Weight	Isoelectric Point	Aliphatic Index	Grand Average of Hydropathicity	Putative Localizatin
GbUBC1	Gbar_A01G001550.1	960	320	36382.37	4.52	68.19	-0.587	Nucleus
GbUBC2	Gbar_A01G004680.1	459	153	17247.93	6.74	92.55	-0.317	Cytoplasm. Nucleus
GbUBC3	Gbar_A01G005430.4	1659	553	61727.64	6.44	70.29	-0.444	Nucleus
GbUBC4	Gbar_A01G010590.1	537	179	20455.47	8.59	82.74	-0.264	Nucleus
GbUBC5	Gbar_A01G014530.1	477	159	17842.35	4.67	70.44	-0.465	Nucleus
GbUBC6	Gbar_A01G016510.1	432	144	16132.71	8.69	81.32	-0.283	Nucleus
GbUBC7	Gbar_A01G020060.3	432	144	16660.01	6.52	71.04	-0.674	Nucleus
GbUBC8	Gbar_A02G000160.1	444	148	16492.06	7.72	77.16	-0.272	Nucleus
GbUBC9	Gbar_A02G010830.1	444	148	16520.11	7.71	75.81	-0.308	Nucleus
GbUBC10	Gbar_A02G018000.1	483	161	18331.78	7.69	73.29	-0.501	Nucleus
GbUBC11	Gbar_A03G002590.2	552	184	21030.65	4.42	65.71	-0.647	Cytoplasm
GbUBC12	Gbar_A03G004830.1	498	166	18682.16	4.94	77.47	-0.375	Nucleus
GbUBC13	Gbar_A03G013550.3	471	157	17929.54	4.96	78.15	-0.459	Nucleus
GbUBC14	Gbar_A03G017430.3	1464	488	54293.43	4.71	72.3	-0.528	Nucleus
GbUBC15	Gbar_A04G000140.1	444	148	16475.92	7.72	74.53	-0.333	Nucleus
GbUBC16	Gbar_A04G003790.1	459	153	17210.94	6.74	91.9	-0.295	Cytoplasm. Nucleus
GbUBC17	Gbar_A04G013530.1	3279	1093	120914.28	4.64	72.8	-0.672	Nucleus
GbUBC18	Gbar_A04G015330.1	477	159	18062.64	4.99	72.83	-0.511	Nucleus
GbUBC19	Gbar_A05G009350.1	498	166	18511.87	4.71	71.57	-0.371	Nucleus
GbUBC20	Gbar_A05G018740.1	483	161	18420	8.36	77.52	-0.459	Nucleus
GbUBC21	Gbar_A05G020330.3	480	160	18021.46	7.69	77.31	-0.426	Nucleus
GbUBC22	Gbar_A05G027100.1	444	148	16345.7	6.4	74.53	-0.378	Nucleus
GbUBC23	Gbar_A05G029480.1	480	160	17647.02	5.65	73.75	-0.255	Nucleus
GbUBC24	Gbar_A05G041600.1	573	191	21308.01	5.39	69.95	-0.452	Nucleus
GbUBC25	Gbar_A06G006360.2	1560	520	57902.02	7.58	70.87	-0.829	Nucleus
GbUBC26	Gbar_A06G009810.1	471	157	17739.51	8.69	79.62	-0.32	Nucleus
GbUBC27	Gbar_A06G010890.2	525	175	18865.42	7.74	77.6	-0.103	Nucleus
GbUBC28	Gbar_A06G013380.1	2763	921	103287.49	5.14	81.22	-0.301	Chloroplast. Nucleus
GbUBC29	Gbar_A06G015420.1	708	236	26833.03	9.36	72.63	-0.464	Nucleus
GbUBC30	Gbar_A07G002770.1	471	157	17771.57	8.7	76.5	-0.322	Nucleus
GbUBC31	Gbar_A07G006630.1	540	180	20894.64	4.55	77.33	-0.577	Nucleus
GbUBC32	Gbar_A07G010190.1	498	166	18702.24	5.2	77.47	-0.39	Nucleus
GbUBC33	Gbar_A07G012900.1	534	178	19616.21	5.6	72.36	-0.354	Nucleus
GbUBC34	Gbar_A08G003010.1	444	148	16465.94	7.75	74.53	-0.347	Nucleus
GbUBC35	Gbar_A08G011230.1	444	148	16575.15	7.72	75.81	-0.33	Nucleus
GbUBC36	Gbar_A08G016540.1	480	160	18205.69	9.01	71.88	-0.631	Nucleus
GbUBC37	Gbar_A08G023610.1	456	152	17412.67	5.37	67.43	-0.566	Cytoplasm. Nucleus
GbUBC38	Gbar_A09G012500.1	339	113	12544.57	6.81	82.92	-0.187	Nucleus
GbUBC39	Gbar_A09G018180.1	555	185	21041.53	4.48	70.05	-0.661	Cytoplasm

GbUBC40	Gbar_A09G019350.1	444	148	16590.12	7.72	73.18	-0.38	Nucleus
GbUBC41	Gbar_A09G022840.1	444	148	16489.06	7.72	77.16	-0.264	Nucleus
GbUBC42	Gbar_A09G025070.1	951	317	36254.74	9.44	76.59	-0.454	Nucleus
GbUBC43	Gbar_A09G025130.1	924	308	34570.22	5.96	75.68	-0.53	Endoplasmic reticulum
GbUBC44	Gbar_A10G002820.1	2742	914	102465.07	4.91	77.26	-0.369	Nucleus
GbUBC45	Gbar_A10G005430.1	690	230	26050.21	9.33	68.61	-0.428	Nucleus
GbUBC46	Gbar_A10G016430.1	444	148	16448.01	7.72	77.16	-0.275	Nucleus
GbUBC47	Gbar_A10G018400.1	918	306	34114.76	5.87	75.59	-0.503	Nucleus
GbUBC48	Gbar_A11G000970.1	651	217	24751.45	9.37	71.01	-0.444	Nucleus
GbUBC49	Gbar_A11G001850.1	552	184	21028.67	4.43	66.25	-0.633	Cytoplasm
GbUBC50	Gbar_A11G001980.1	444	148	16532.13	7.72	76.49	-0.295	Nucleus
GbUBC51	Gbar_A11G003890.1	513	171	19007.72	5.19	90.76	-0.197	Nucleus
GbUBC52	Gbar_A11G004570.1	549	183	20983.05	7.66	77.21	-0.479	Nucleus
GbUBC53	Gbar_A11G009790.1	444	148	16548.13	7.72	75.81	-0.307	Nucleus
GbUBC54	Gbar_A11G017860.1	444	148	16642.27	7.65	86.96	-0.178	Nucleus
GbUBC55	Gbar_A11G019160.1	549	183	20895.96	8.29	77.21	-0.462	Nucleus
GbUBC56	Gbar_A11G020100.2	480	160	17980.49	8.43	70.12	-0.549	Nucleus
GbUBC57	Gbar_A11G024410.3	456	152	17348.56	5.37	70	-0.576	Cytoplasm. Nucleus
GbUBC58	Gbar_A11G030160.1	1950	650	72357.1	5.29	82	-0.297	Nucleus
GbUBC59	Gbar_A11G030910.2	459	153	17205.85	6.74	92.55	-0.319	Cytoplasm. Nucleus
GbUBC60	Gbar_A11G032790.1	804	268	29025.24	8.79	84.1	-0.458	Nucleus
GbUBC61	Gbar_A11G033860.1	438	146	16630.92	6.42	73.36	-0.552	Nucleus
GbUBC62	Gbar_A11G033870.1	438	146	16715	5.93	73.36	-0.565	Nucleus
GbUBC63	Gbar_A11G033880.1	438	146	16472.7	6.07	72.67	-0.522	Nucleus
GbUBC64	Gbar_A12G003670.1	444	148	16644.2	8.33	81.08	-0.329	Nucleus
GbUBC65	Gbar_A12G006330.1	483	161	18366.86	7.68	76.27	-0.452	Nucleus
GbUBC66	Gbar_A12G006800.1	549	183	21130.12	6.31	82.57	-0.53	Nucleus
GbUBC67	Gbar_A12G017830.1	456	152	17249.43	5.1	70	-0.549	Cytoplasm. Nucleus
GbUBC68	Gbar_A12G018720.2	456	152	17362.59	5.37	70.66	-0.574	Cytoplasm. Nucleus
GbUBC69	Gbar_A12G020550.1	582	194	21289.18	4.75	88.97	-0.24	Nucleus
GbUBC70	Gbar_A12G026340.1	444	148	16506.09	7.71	75.81	-0.309	Nucleus
GbUBC71	Gbar_A12G029100.1	552	184	21119.74	4.41	65.65	-0.642	Cytoplasm
GbUBC72	Gbar_A13G024300.1	498	166	18667.15	5.07	78.07	-0.364	Nucleus
GbUBC73	Gbar_D01G001720.1	1398	466	52840.23	4.7	69.85	-0.457	Nucleus
GbUBC74	Gbar_D01G003810.1	459	153	17285.98	6.74	95.1	-0.32	Cytoplasm. Nucleus
GbUBC75	Gbar_D01G005660.5	1659	553	61668.63	6.38	72.06	-0.436	Nucleus
GbUBC76	Gbar_D01G011150.1	483	161	18424.01	8.28	75.71	-0.474	Nucleus
GbUBC77	Gbar_D01G015390.1	477	159	17941.48	4.77	70.44	-0.491	Nucleus
GbUBC78	Gbar_D01G021190.1	438	146	16602.87	6.2	70	-0.608	Nucleus
GbUBC79	Gbar_D02G000270.1	444	148	16522.09	7.72	76.49	-0.289	Nucleus
GbUBC80	Gbar_D02G015200.1	477	159	18045.58	4.87	73.46	-0.516	Nucleus
GbUBC81	Gbar_D02G019250.6	1092	364	40735.21	6.11	77.09	-0.399	Nucleus
GbUBC82	Gbar_D03G015870.2	672	224	25611.17	4.64	74.42	-0.418	Cytoplasm

GbUBC83	Gbar_D04G000310.1	573	191	21299.06	5.44	69.48	-0.445	Nucleus
GbUBC84	Gbar_D04G018200.1	3345	1115	123400.86	4.55	73.82	-0.66	Nucleus
GbUBC85	Gbar_D04G020140.1	477	159	18068.62	5.1	70.38	-0.543	Nucleus
GbUBC86	Gbar_D05G009810.1	498	166	18542.93	4.77	72.17	-0.336	Nucleus
GbUBC87	Gbar_D05G019440.1	483	161	18500.04	8.36	73.85	-0.497	Nucleus
GbUBC88	Gbar_D05G021000.1	480	160	18084.55	8.44	77.31	-0.437	Nucleus
GbUBC89	Gbar_D05G027950.1	444	148	16348.66	5.74	76.49	-0.341	Nucleus
GbUBC90	Gbar_D05G030260.1	573	191	21288.93	5.19	66.91	-0.464	Nucleus
GbUBC91	Gbar_D05G035090.2	459	153	17263.93	6.74	91.9	-0.334	Cytoplasm. Nucleus
GbUBC92	Gbar_D06G006640.1	1560	520	58018.47	8.74	73.87	-0.798	Nucleus
GbUBC93	Gbar_D06G010300.1	471	157	17737.5	8.7	80.25	-0.308	Nucleus
GbUBC94	Gbar_D06G012060.1	525	175	18788.26	8.57	76.46	-0.157	Nucleus
GbUBC95	Gbar_D06G014120.1	2769	923	103532.67	5.08	80.93	-0.312	Chloroplast. Nucleus
GbUBC96	Gbar_D06G016040.1	708	236	26838	9.06	72.2	-0.458	Nucleus
GbUBC97	Gbar_D07G006930.1	384	128	14650.33	4.21	73.05	-0.576	Nucleus
GbUBC98	Gbar_D07G010570.2	498	166	18679.2	5.07	77.47	-0.392	Nucleus
GbUBC99	Gbar_D07G013300.1	513	171	19456.34	5.41	80.41	-0.228	Nucleus
GbUBC100	Gbar_D08G003120.1	444	148	16491.98	7.72	74.53	-0.334	Nucleus
GbUBC101	Gbar_D08G017260.1	483	161	18124.7	9.45	70.75	-0.543	Nucleus
GbUBC102	Gbar_D09G017910.1	555	185	21042.52	4.44	70.05	-0.661	Cytoplasm
GbUBC103	Gbar_D09G019160.1	444	148	16606.18	7.67	73.18	-0.357	Nucleus
GbUBC104	Gbar_D09G024720.1	966	322	37113.8	9.25	76.93	-0.409	Nucleus
GbUBC105	Gbar_D09G024780.1	924	308	34536.11	5.96	75.68	-0.559	Nucleus
GbUBC106	Gbar_D10G002720.1	2742	914	102651.32	4.91	77.48	-0.375	Nucleus
GbUBC107	Gbar_D10G005260.1	690	230	26130.34	9.33	70.74	-0.44	Nucleus
GbUBC108	Gbar_D10G011720.1	444	148	16448.01	7.72	77.16	-0.27	Nucleus
GbUBC109	Gbar_D10G018560.1	918	306	34173.75	5.95	74.02	-0.556	Nucleus
GbUBC110	Gbar_D11G001330.1	651	217	24780.56	9.49	73.27	-0.418	Nucleus
GbUBC111	Gbar_D11G002190.1	552	184	21014.6	4.38	66.25	-0.63	Cytoplasm
GbUBC112	Gbar_D11G010330.1	393	131	14535.86	6.39	81.91	-0.172	Nucleus
GbUBC113	Gbar_D11G018780.1	444	148	16672.36	7.65	86.96	-0.161	Nucleus
GbUBC114	Gbar_D11G031220.1	1878	626	69815.02	5.3	81.12	-0.304	Nucleus
GbUBC115	Gbar_D11G033560.1	810	270	29246.54	8.79	86.74	-0.449	Nucleus
GbUBC116	Gbar_D11G036910.1	438	146	16625.89	6.91	74.04	-0.588	Nucleus
GbUBC117	Gbar_D11G036920.2	489	163	18417.99	8.42	71.72	-0.543	Nucleus
GbUBC118	Gbar_D12G003560.1	444	148	16585.13	7.67	81.08	-0.309	Nucleus
GbUBC119	Gbar_D12G006400.1	483	161	18352.84	7.68	75.65	-0.465	Nucleus
GbUBC120	Gbar_D12G006820.1	549	183	21175.12	6.16	79.89	-0.542	Nucleus
GbUBC121	Gbar_D12G010490.1	459	153	17219.87	6.74	92.55	-0.318	Cytoplasm. Nucleus
GbUBC122	Gbar_D12G020750.1	582	194	21220.07	4.67	88.97	-0.221	Nucleus
GbUBC123	Gbar_D12G024010.1	552	184	21089.71	4.41	66.2	-0.628	Cytoplasm
GbUBC124	Gbar_D12G026270.1	444	148	16536.11	7.71	75.14	-0.326	Nucleus
GbUBC125	Gbar_D13G024860.1	498	166	18598.04	4.94	78.67	-0.336	Nucleus
