# Supplementary material for: Genome-Wide Identification of the GbUBC Gene Family in Sea-Island Cotton (Gossypium barbadense) and the Active Regulation of Drought Resistance in Cotton by GbUBC23
Source: Int J Mol Sci. 2024 Dec 2;25(23):12948. doi: 10.3390/ijms252312948 (PMC11640981; doi:10.3390/ijms252312948)
Supplement: Supplementary file 1 [file ijms-25-12948-s001.zip › Table S3.pdf]

Table S3 Information on duplication events of GbUBC genes

Gene Name	Gene ID	Gene Name	Gene ID	Ka	Ks	Ka/Ks
GbUBC4	Gbar_A01G010590.1	GbUBC10	Gbar_A02G018000.1	0.071798456	NaN	NaN
GbUBC5	Gbar_A01G014530.1	GbUBC18	Gbar_A04G015330.1	0.05133765	0.52830778	0.097173754
GbUBC2	Gbar_A01G004680.1	GbUBC16	Gbar_A04G003790.1	0.012928259	0.311373379	0.041520116
GbUBC4	Gbar_A01G010590.1	GbUBC20	Gbar_A05G018740.1	0.021569674	0.352151516	0.061251116
GbUBC6	Gbar_A01G016510.1	GbUBC64	Gbar_A12G003670.1	0.040102073	0.496877221	0.080708213
GbUBC5	Gbar_A01G014530.1	GbUBC77	Gbar_D01G015390.1	0.002678574	0.019673259	0.136153052
GbUBC5	Gbar_A01G014530.1	GbUBC85	Gbar_D04G020140.1	0.05126649	0.49379141	0.103822158
GbUBC4	Gbar_A01G010590.1	GbUBC87	Gbar_D05G019440.1	0.024288158	0.36877028	0.065862569
GbUBC10	Gbar_A02G018000.1	GbUBC20	Gbar_A05G018740.1	0.054506737	4.330913659	0.012585505
GbUBC9	Gbar_A02G010830.1	GbUBC35	Gbar_A08G011230.1	0.005838025	0.366241447	0.015940373
GbUBC8	Gbar_A02G000160.1	GbUBC46	Gbar_A10G016430.1	0.008819308	0.516186909	0.017085493
GbUBC9	Gbar_A02G010830.1	GbUBC50	Gbar_A11G001980.1	0.008774168	0.334364139	0.026241354
GbUBC9	Gbar_A02G010830.1	GbUBC70	Gbar_A12G026340.1	0.017613334	1.628049721	0.010818671
GbUBC10	Gbar_A02G018000.1	GbUBC65	Gbar_A12G006330.1	0.018877401	0.72599408	0.026002142
GbUBC10	Gbar_A02G018000.1	GbUBC87	Gbar_D05G019440.1	0.063085668	3.288860072	0.019181621
GbUBC9	Gbar_A02G010830.1	GbUBC103	Gbar_D09G019160.1	0.020584482	1.473416068	0.013970584
GbUBC8	Gbar_A02G000160.1	GbUBC108	Gbar_D10G011720.1	0.005865133	0.517405391	0.011335662
GbUBC9	Gbar_A02G010830.1	GbUBC124	Gbar_D12G026270.1	0.014645327	1.341932808	0.010913607
GbUBC12	Gbar_A03G004830.1	GbUBC19	Gbar_A05G009350.1	0.056075931	0.341606647	0.164153512
GbUBC12	Gbar_A03G004830.1	GbUBC32	Gbar_A07G010190.1	0.010371816	0.354785829	0.02923402
GbUBC12	Gbar_A03G004830.1	GbUBC72	Gbar_A13G024300.1	0.010365097	0.449573053	0.023055423
GbUBC11	Gbar_A03G002590.2	GbUBC82	Gbar_D03G015870.2	0.00461008	0.044065535	0.104618718
GbUBC12	Gbar_A03G004830.1	GbUBC86	Gbar_D05G009810.1	0.05048386	0.358267547	0.140911061
GbUBC12	Gbar_A03G004830.1	GbUBC98	Gbar_D07G010570.2	0.007765384	0.369564632	0.02101225
GbUBC11	Gbar_A03G002590.2	GbUBC123	Gbar_D12G024010.1	0.046223156	0.584536394	0.07907661
GbUBC12	Gbar_A03G004830.1	GbUBC125	Gbar_D13G024860.1	0.018230064	0.386174212	0.04720684
GbUBC11	Gbar_A03G002590.2	GbUBC71	Gbar_A12G029100.1	0.048672138	0.566130562	0.085973345
GbUBC18	Gbar_A04G015330.1	GbUBC77	Gbar_D01G015390.1	0.048448575	0.491555139	0.098561832
GbUBC18	Gbar_A04G015330.1	GbUBC85	Gbar_D04G020140.1	0.010820747	0.070050881	0.154469816
GbUBC19	Gbar_A05G009350.1	GbUBC32	Gbar_A07G010190.1	0.064498091	0.354785829	0.181794441
GbUBC20	Gbar_A05G018740.1	GbUBC65	Gbar_A12G006330.1	0.053076803	NaN	NaN
GbUBC19	Gbar_A05G009350.1	GbUBC72	Gbar_A13G024300.1	0.056088417	0.355823085	0.157630069
GbUBC24	Gbar_A05G041600.1	GbUBC83	Gbar_D04G000310.1	0.01026632	0.042980509	0.238859898
GbUBC19	Gbar_A05G009350.1	GbUBC86	Gbar_D05G009810.1	0.012959286	0.009245109	1.401744988
GbUBC20	Gbar_A05G018740.1	GbUBC87	Gbar_D05G019440.1	0.008017894	0.018958355	0.422921375
GbUBC22	Gbar_A05G027100.1	GbUBC89	Gbar_D05G027950.1	0.008810674	0.040482001	0.217644232
GbUBC24	Gbar_A05G041600.1	GbUBC90	Gbar_D05G030260.1	0.056419777	0.515592274	0.10942712
GbUBC19	Gbar_A05G009350.1	GbUBC98	Gbar_D07G010570.2	0.061696968	0.340292624	0.181305627
GbUBC19	Gbar_A05G009350.1	GbUBC125	Gbar_D13G024860.1	0.061641819	0.3273205	0.188322512
GbUBC26	Gbar_A06G009810.1	GbUBC30	Gbar_A07G002770.1	0.009655306	0.442726199	0.021808752
GbUBC28	Gbar_A06G013380.1	GbUBC44	Gbar_A10G002820.1	0.140043443	0.549174862	0.255007017

GbUBC26	Gbar_A06G009810.1	GbUBC93	Gbar_D06G010300.1	0.005521074	0.048054864	0.114891053
GbUBC28	Gbar_A06G013380.1	GbUBC95	Gbar_D06G014120.1	0.017178569	0.03245999	0.529222869
GbUBC32	Gbar_A07G010190.1	GbUBC72	Gbar_A13G024300.1	0.015615398	0.431333307	0.036202625
GbUBC32	Gbar_A07G010190.1	GbUBC86	Gbar_D05G009810.1	0.056050974	0.371755364	0.150773813
GbUBC30	Gbar_A07G002770.1	GbUBC93	Gbar_D06G010300.1	0.015239499	0.441309997	0.034532413
GbUBC31	Gbar_A07G006630.1	GbUBC97	Gbar_D07G006930.1	0.092663463	0.10496948	0.882765758
GbUBC32	Gbar_A07G010190.1	GbUBC98	Gbar_D07G010570.2	0.002581758	0.066499228	0.038823882
GbUBC33	Gbar_A07G012900.1	GbUBC99	Gbar_D07G013300.1	0.073125791	0.136981977	0.533835129
GbUBC32	Gbar_A07G010190.1	GbUBC125	Gbar_D13G024860.1	0.018245904	0.400025671	0.045611833
GbUBC35	Gbar_A08G011230.1	GbUBC53	Gbar_A11G009790.1	0.014728014	1.800896704	0.008178156
GbUBC36	Gbar_A08G016540.1	GbUBC101	Gbar_D08G017260.1	0.112427693	0.165253875	0.680333172
GbUBC34	Gbar_A08G003010.1	GbUBC100	Gbar_D08G003120.1	0.002919712	0.030358276	0.096175149
GbUBC35	Gbar_A08G011230.1	GbUBC112	Gbar_D11G010330.1	0.009947645	1.937823441	0.005133412
GbUBC43	Gbar_A09G025130.1	GbUBC47	Gbar_A10G018400.1	0.086812897	0.616329238	0.140854744
GbUBC42	Gbar_A09G025070.1	GbUBC104	Gbar_D09G024720.1	0.014954614	0.00482123	3.101825403
GbUBC43	Gbar_A09G025130.1	GbUBC105	Gbar_D09G024780.1	0.014240371	0.047997182	0.29669181
GbUBC43	Gbar_A09G025130.1	GbUBC109	Gbar_D10G018560.1	0.090825211	0.600300133	0.151299669
GbUBC44	Gbar_A10G002820.1	GbUBC95	Gbar_D06G014120.1	0.135670084	0.538588036	0.251899549
GbUBC47	Gbar_A10G018400.1	GbUBC105	Gbar_D09G024780.1	0.07876329	0.577414386	0.136406872
GbUBC47	Gbar_A10G018400.1	GbUBC109	Gbar_D10G018560.1	0.014272552	0.033986397	0.419948971
GbUBC46	Gbar_A10G016430.1	GbUBC108	Gbar_D10G011720.1	0.00292398	0.051040097	0.057287906
GbUBC52	Gbar_A11G004570.1	GbUBC55	Gbar_A11G019160.1	0.039904636	0.426904628	0.093474358
GbUBC55	Gbar_A11G019160.1	GbUBC66	Gbar_A12G006800.1	0.108615576	0.497470663	0.218335641
GbUBC51	Gbar_A11G003890.1	GbUBC69	Gbar_A12G020550.1	0.158871826	0.364263476	0.436145362
GbUBC52	Gbar_A11G004570.1	GbUBC66	Gbar_A12G006800.1	0.108592678	0.513820084	0.211343778
GbUBC48	Gbar_A11G000970.1	GbUBC70	Gbar_A12G026340.1	0.008742207	0.539075783	0.016217028
GbUBC61	Gbar_A11G033860.1	GbUBC78	Gbar_D01G021190.1	0.024275191	0.292965226	0.082860316
GbUBC48	Gbar_A11G000970.1	GbUBC103	Gbar_D09G019160.1	0.008740084	0.53974184	0.016193083
GbUBC48	Gbar_A11G000970.1	GbUBC110	Gbar_D11G001330.1	0.007977473	0.041993475	0.189969338
GbUBC49	Gbar_A11G001850.1	GbUBC111	Gbar_D11G002190.1	0.002302381	0.044000886	0.052325786
GbUBC53	Gbar_A11G009790.1	GbUBC112	Gbar_D11G010330.1	0.016690536	0.045772066	0.364644579
GbUBC54	Gbar_A11G017860.1	GbUBC113	Gbar_D11G018780.1	0.002932555	0.019803131	0.148085427
GbUBC50	Gbar_A11G001980.1	GbUBC112	Gbar_D11G010330.1	0.009936662	1.973005117	0.005036308
GbUBC55	Gbar_A11G019160.1	GbUBC120	Gbar_D12G006820.1	0.100379265	0.500339764	0.200622202
GbUBC51	Gbar_A11G003890.1	GbUBC122	Gbar_D12G020750.1	0.160433015	0.385957443	0.415675403
GbUBC49	Gbar_A11G001850.1	GbUBC123	Gbar_D12G024010.1	0.03653511	0.416522609	0.08771459
GbUBC52	Gbar_A11G004570.1	GbUBC120	Gbar_D12G006820.1	0.103047627	0.533171315	0.193273013
GbUBC48	Gbar_A11G000970.1	GbUBC124	Gbar_D12G026270.1	0.005815391	0.479809803	0.012120201
GbUBC61	Gbar_A11G033860.1	GbUBC116	Gbar_D11G036910.1	0.01512148	0.222199362	0.068053661
GbUBC65	Gbar_A12G006330.1	GbUBC87	Gbar_D05G019440.1	0.061640581	3.808720457	0.016184065
GbUBC70	Gbar_A12G026340.1	GbUBC110	Gbar_D11G001330.1	0.008750707	0.516088374	0.01695583
GbUBC69	Gbar_A12G020550.1	GbUBC122	Gbar_D12G020750.1	0.002254793	0.014652481	0.153884737
GbUBC66	Gbar_A12G006800.1	GbUBC120	Gbar_D12G006820.1	0.00706719	0.008208016	0.861010734

GbUBC70	Gbar_A12G026340.1	GbUBC124	Gbar_D12G026270.1	0.002903464	0.063250807	0.045903977
GbUBC72	Gbar_A13G024300.1	GbUBC86	Gbar_D05G009810.1	0.04775093	0.343263764	0.139108567
GbUBC72	Gbar_A13G024300.1	GbUBC98	Gbar_D07G010570.2	0.012990149	0.384259036	0.03380571
GbUBC72	Gbar_A13G024300.1	GbUBC125	Gbar_D13G024860.1	0.012978909	0.037478523	0.346302579
GbUBC77	Gbar_D01G015390.1	GbUBC85	Gbar_D04G020140.1	0.04838158	0.458436114	0.105536144
GbUBC78	Gbar_D01G021190.1	GbUBC116	Gbar_D11G036910.1	0.033601955	0.399850053	0.084036389
GbUBC82	Gbar_D03G015870.2	GbUBC123	Gbar_D12G024010.1	0.04505503	0.553586154	0.081387567
GbUBC82	Gbar_D03G015870.2	GbUBC71	Gbar_A12G029100.1	0.047503013	0.535990039	0.088626671
GbUBC83	Gbar_D04G000310.1	GbUBC90	Gbar_D05G030260.1	0.050321072	0.479535567	0.104937101
GbUBC86	Gbar_D05G009810.1	GbUBC98	Gbar_D07G010570.2	0.053284719	0.356866538	0.149312735
GbUBC86	Gbar_D05G009810.1	GbUBC125	Gbar_D13G024860.1	0.05323742	0.315090158	0.168959324
GbUBC98	Gbar_D07G010570.2	GbUBC125	Gbar_D13G024860.1	0.015612012	0.384640517	0.040588579
GbUBC105	Gbar_D09G024780.1	GbUBC109	Gbar_D10G018560.1	0.081933704	0.567192762	0.144454778
GbUBC103	Gbar_D09G019160.1	GbUBC110	Gbar_D11G001330.1	0.00874858	0.496877221	0.017607127
GbUBC111	Gbar_D11G002190.1	GbUBC123	Gbar_D12G024010.1	0.038954467	0.387392734	0.100555492
GbUBC110	Gbar_D11G001330.1	GbUBC124	Gbar_D12G026270.1	0.005821033	0.458713185	0.012689919
GbUBC123	Gbar_D12G024010.1	GbUBC71	Gbar_A12G029100.1	0.00230592	0.043744175	0.052713769
