# Supplementary material for: Genome-Wide Identification of the GbUBC Gene Family in Sea-Island Cotton (Gossypium barbadense) and the Active Regulation of Drought Resistance in Cotton by GbUBC23
Source: Int J Mol Sci. 2024 Dec 2;25(23):12948. doi: 10.3390/ijms252312948 (PMC11640981; doi:10.3390/ijms252312948)
Supplement: Supplementary file 1 [file ijms-25-12948-s001.zip › Table S4.pdf]

Table S4 Primer sequences used in this study

| Name | Sequence |
|---|---|
| GbUBC23-qF | CAGCTGCCGCTTCATCTAAAC |
| GbUBC23-qR | CCGACACTCCAAGATCACCT |
| GbUBC23-1304-F | cgggggactcttgaccatggaaATGGAAGTCCGACCCAATC |
| GbUBC23-1304-R | ctcctttactagtcagatctttGTAGGGATGAGCTAGCAGA |
| GhUBC23-TRV2-F | tgagtaaggttaccgaattcTTTGGTGGAAAAGCGTATGAGA |
| GhUBC23-TRV2-R | gcgtgagctcggtaccTCTACCATATCAATATCATGCT |
