# Supplementary figures and images for: Genome-Wide Identification of the GbUBC Gene Family in Sea-Island Cotton (Gossypium barbadense) and the Active Regulation of Drought Resistance in Cotton by GbUBC23
Source: Int J Mol Sci. 2024 Dec 2;25(23):12948. doi: 10.3390/ijms252312948 (PMC11640981; doi:10.3390/ijms252312948)

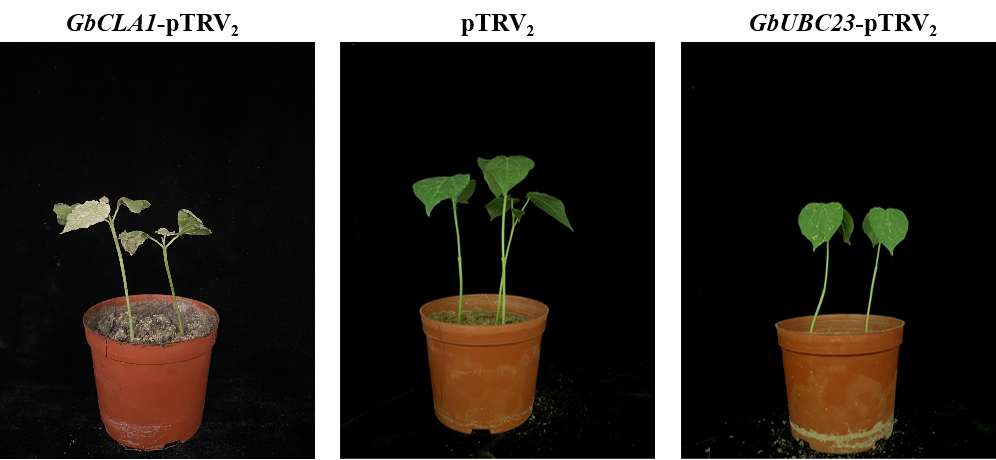

Supplement: Supplementary file 1 [file ijms-25-12948-s001.zip › Figure S1.tif]
